# Galectin-3 enhances neutrophil motility and extravasation into the airways during *Aspergillus fumigatus* infection

**DOI:** 10.1371/journal.ppat.1008741

**Published:** 2020-08-04

**Authors:** Brendan D. Snarr, Guillaume St-Pierre, Benjamin Ralph, Mélanie Lehoux, Yukiko Sato, Ann Rancourt, Takahiro Takazono, Shane R. Baistrocchi, Rachel Corsini, Matthew P. Cheng, Michele Sugrue, Lindsey R. Baden, Koichi Izumikawa, Hiroshi Mukae, John R. Wingard, Irah L. King, Maziar Divangahi, Masahiko S. Satoh, Bryan G. Yipp, Sachiko Sato, Donald C. Sheppard

**Affiliations:** 1 Department of Microbiology and Immunology, McGill University, Montréal, Canada; 2 Infectious Diseases and Immunity in Global Health Program, Centre for Translational Biology, Research Institute of the McGill University Health Centre, Montréal, Canada; 3 McGill Interdisciplinary Initiative in Infection and Immunity, Montréal, Canada; 4 Laboratory of Glycobiology and Bioimaging, Research Centre for Infectious Diseases, Research Centre of CHU de Québec, Faculty of Medicine, Laval University, Québec City, Canada; 5 Laboratory of DNA Damage Responses and Bioimaging, CHU de Québec, Faculty of Medicine, Laval University, Québec city, Canada; 6 Department of Infectious Diseases, Nagasaki University Graduate School of Biomedical Sciences, Nagasaki, Japan; 7 Department of Respiratory Medicine, Nagasaki University Graduate School of Biomedical Sciences, Nagasaki, Japan; 8 Division of Infectious Diseases and Department of Medical Microbiology, McGill University Health Centre, Montréal, Canada; 9 University of Florida College of Medicine, Gainsville, Florida, United States of America; 10 Harvard University & Brigham & Women’s Hospital, Boston, Massachusetts, United States of America; 11 Meakins-Christie Laboratories, Department of Medicine, Department of Pathology, McGill International TB Centre, McGill University Health Centre, Montréal, Canada; 12 Calvin, Phoebe and Joan Snyder Institute for Chronic Diseases, Cumming School of Medicine, University of Calgary, Calgary, Canada; Tulane University School of Medicine, UNITED STATES

## Abstract

*Aspergillus fumigatus* is an opportunistic mold that infects patients who are immunocompromised or have chronic lung disease, causing significant morbidity and mortality in these populations. While the factors governing the host response to *A*. *fumigatus* remain poorly defined, neutrophil recruitment to the site of infection is critical to clear the fungus. Galectin-3 is a mammalian β-galactose-binding lectin with both antimicrobial and immunomodulatory activities, however the role of galectin-3 in the defense against molds has not been studied. Here we show that galectin-3 expression is markedly up-regulated in mice and humans with pulmonary aspergillosis. Galectin-3 deficient mice displayed increased fungal burden and higher mortality during pulmonary infection. In contrast to previous reports with pathogenic yeast, galectin-3 exhibited no antifungal activity against *A*. *fumigatus in vitro*. Galectin-3 deficient mice exhibited fewer neutrophils in their airways during infection, despite normal numbers of total lung neutrophils. Intravital imaging studies confirmed that galectin-3 was required for normal neutrophil migration to the airspaces during fungal infection. Adoptive transfer experiments demonstrated that stromal rather than neutrophil-intrinsic galectin-3 was necessary for normal neutrophil entry into the airspaces. Live cell imaging studies revealed that extracellular galectin-3 directly increases neutrophil motility. Taken together, these data demonstrate that extracellular galectin-3 facilitates recruitment of neutrophils to the site of *A*. *fumigatus* infection, and reveals a novel role for galectin-3 in host defense against fungal infections.

## Author summary

The environmental mold *Aspergillus fumigatus* commonly causes lung infections in people with impaired immunity or those suffering from a chronic lung disease. While neutrophils are a key cell type necessary for the eradication of this infection, the precise mechanism of their recruitment to the site of infection remains incompletely understood. Here we show that the secreted mammalian protein galectin-3 plays an important role in helping neutrophils reaching the fungus within the airways. We found that both mice and humans produce galectin-3 when infected with *A*. *fumigatus*, and mice lacking galectin-3 were more susceptible to infection than normal mice. Galectin-3-deficient mice had impaired neutrophil recruitment to the site of infection. In the absence of galectin-3, neutrophils exhibited reduced motility in mouse lungs and in tissue culture. Our study offers insights into the mechanisms underlying the recruitment of neutrophils to the airways during *A*. *fumigatus* infection and reveals a new role for galectin-3 in increasing neutrophil motility.

## Introduction

*Aspergillus fumigatus* is an opportunistic fungal pathogen that commonly infects the respiratory tract of both immunocompromised patients and patients with chronic lung diseases such as cystic fibrosis [1–3]. Pulmonary *A*. *fumigatus* infection manifests as a necrotizing pneumonia, which can disseminate via the bloodstream to distal organs such as the brain. Both clinical and experimental studies have shown that neutrophils play a vital role in the host defense against *A*. *fumigatus* [1,2]. The mortality rate of invasive aspergillosis remains between 30 and 95% [3], despite the use of available antifungal therapies. There is thus an urgent need for novel treatment approaches for invasive fungal *A*. *fumigatus* infections. One such approach is to enhance innate immune responses to this filamentous fungus.

Galectin-3 is a soluble mammalian lectin that has been implicated in the immune response against diverse microbial infections [4,5], including bacteria [6,7], parasites [8–10], and pathogenic yeast [11–13]. Galectin-3 is expressed in the cytosol of both immune and stromal cells, and is released into the extracellular space through active secretion [14,15] and following cellular injury [16].

Galectin-3 is composed of a single C-terminal carbohydrate recognition domain (CRD), which binds to β-galactoside-containing carbohydrates [17] and an N-terminal domain. The N-terminal domain of galectin-3 is an intrinsically disordered tandem-repeat region [18,19] that can interact with the N-terminal domain of other galectin-3 monomers, allowing for multimerization [20–23]. Galectin-3 multimerization is a dynamic process, resulting in the formation of oligomeric structures [20–23]. The CRDs remain exposed following oligomerization, allowing them to bind and cross-link ligands, resulting in a diverse range of outcomes depending on the ligands and cell types involved (reviewed in ref. [24]). During infection, galectin-3 has been reported to mediate host defense against microbes through multiple mechanisms including binding to, and directly killing pathogens, mediating pathogen detection through cross-linking canonical immune receptors, and/or enhancing immune cell recruitment [8,14,25–27].

The role of galectin-3 in host defense against fungal infections varies by species. Galectin-3 deficient mice are more resistant to infection with the intracellular pathogen *Histoplasma caspulatum*, which has been linked to increased production of interleukin (IL)-17-associated cytokines by dendritic cells in these mice [28]. Conversely, galectin-3 deficient mice exhibit increased susceptibility to infection with the pathogenic basidiomycetous yeast *Cryptococcus neoformans* [11]. Galectin-3 was found to bind to and lyse extracellular vesicles of *C*. *neoformans*, resulting in direct fungal killing [11]. Galectin-3 binds to cell wall glycans of the pathogenic yeast *Candida albicans* and is also directly fungicidal via an unknown mechanism [25]. Two studies examining the susceptibility of galectin-3 deficient mice to *C*. *albicans* infection have reported conflicting results with both increased and decreased survival of these animals following fungal challenge [12,29]. The role of galectin-3 in the pathogenesis of *Aspergillus* or other mold infections has not been studied.

In light of these reports, we hypothesized that galectin-3 plays a role in the innate immune response to *A*. *fumigatus* infection. High levels of extracellular galectin-3 were found in the serum of mice infected with *A*. *fumigatus*, as well as human patients with pulmonary aspergillosis. Galectin-3 deficient mice were more susceptible to pulmonary *A*. *fumigatus* infection and exhibited reduced neutrophil migration to the airspaces following fungal challenge. Intravital imaging confirmed that neutrophils exhibited reduced motility within the lungs of galectin-3 deficient mice. Adoptive transfer experiments revealed that stromal, rather than neutrophil-derived galectin-3 was required for effective neutrophil recruitment to the airways, and recombinant galectin-3 enhanced the motility of human neutrophils *in vitro*, suggesting that extracellular galectin-3 plays a direct role in stimulating neutrophil motility.

## Results

### Galectin-3 is released in response to *A*. *fumigatus in vivo*

**Fig 1:**
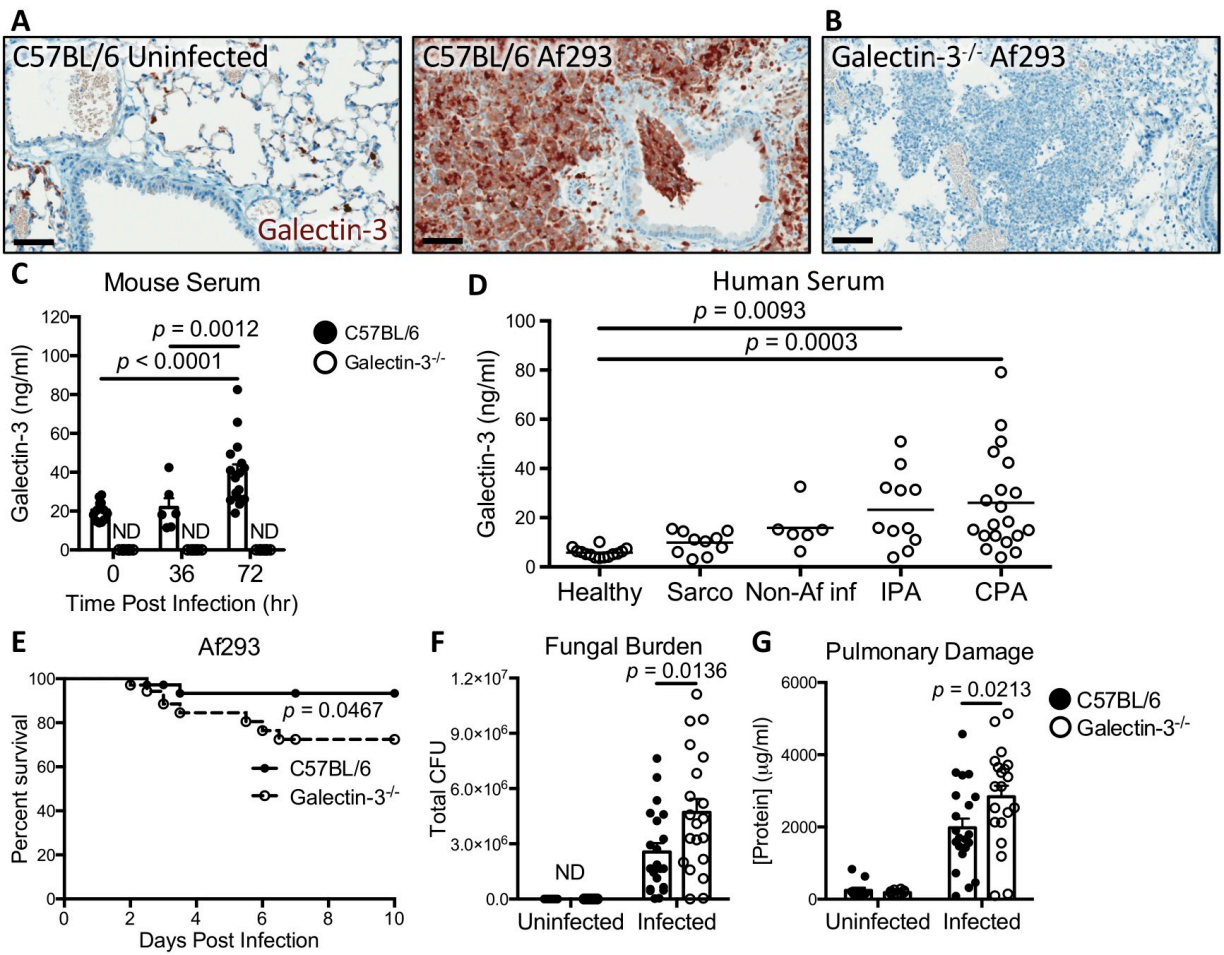
Galectin-3 protects against *Aspergillus fumigatus* infection. **(A)** Pulmonary tissue sections from C57BL/6 mice uninfected or infected with Af293 conidia and stained for galectin-3 (brown) 36 hours post-infection. **(B)** Pulmonary tissue section from a galectin-3 deficient mouse stained for galectin-3 as in (A). Scale bar = 60 μm. **(C)** Galectin-3 quantification by EIA in sera from the indicated mouse strains. *n* = 15, 6 and 16 for C57BL/6 and 8, 6, and 8 for galectin-3 deficient mice at 0, 36, and 72 hours post-infection, respectively. ND: not detected. **(D)** Galectin-3 quantification by EIA in the sera of healthy humans, as well as patients with sarcoidosis (sarco), non-*Aspergillus* pulmonary infections (Non-Af inf), invasive pulmonary aspergillosis (IPA), and chronic pulmonary aspergillosis (CPA). *n* = 14, 10, 6, 11, and 20 for Healthy, Sarco, Non-Af inf, IPA, and CPA subjects, respectively. **(E)** Survival of C57BL/6 and galectin-3 deficient mice infected with Af293 conidia. *n* = 36 C57BL/6 and 35 galectin-3 deficient mice from 4 independent experiments. **(F)** Pulmonary fungal burden and **(G)** pulmonary injury as a function of total protein content in the BAL fluid at 36 hours post-infection with Af293 conidia. *n* = 7 uninfected and 21 infected C57BL/6, and 7 uninfected and 20 infected galectin-3 deficient mice from 3 independent experiments for (F). *n* = 11 uninfected and 21 infected C57BL/6, and 10 uninfected and 20 infected galectin-3 deficient mice from 3 independent experiments for (G). 2-way ANOVA for (C), (F), and (G), or 1-way ANOVA for (D), all with Sidak’s multiple comparison post-test; Mantel-Cox log rank test for (E).

To determine if galectin-3 is involved in the immune response against *A*. *fumigatus*, both wild-type and galectin-3 deficient mice were infected intratracheally with *A*. *fumigatus* conidia and their lungs were harvested at 36 hours post infection for galectin-3 immunostaining. Rare galectin-3-staining cells were found within the airspaces of uninfected wild-type mice (Fig 1A). In contrast, intense galectin-3 staining of leukocytes and the pulmonary epithelium was observed in infected wild-type mice (Fig 1A). No staining of pulmonary tissues was seen in galectin-3 deficient mice (Fig 1B), confirming the specificity of the anti-galectin-3 antibody.

To identify the cellular source of galectin-3 in infected mouse lungs, pulmonary tissues were enzymatically digested, stained for intracellular galectin-3 and analyzed by flow cytometry (S1 Fig). Galectin-3 was detected in all cell types analyzed, with the highest signals seen in alveolar macrophages, followed by inflammatory monocytes, eosinophils, and neutrophils (S1B, S1C and S1D Fig). Intracellular galectin-3 signal changed minimally in response to infection, with a significant reduction of galectin-3 mean fluorescent intensity (MFI) noted only in alveolar macrophages (S1C Fig). Almost all immune cells were galectin-3-positive, both pre- and post-infection, with an increase in the % positive cells seen only in inflammatory monocytes (83% to 99% galectin-3 positive), and endothelial cells (26% to 59% galectin-3 positive) (S1D Fig). Given the relative abundance of each cell type (S1E Fig), neutrophils and inflammatory monocytes accounted for the largest pools of galectin-3 in infected lungs.

To determine if extracellular galectin-3 levels were increased during *A*. *fumigatus* infection, the concentration of galectin-3 in serum from infected and uninfected mice was measured by ELISA. Galectin-3 levels were elevated in the serum of wild-type mice after 72 hours after infection (Fig 1C). Collectively these data suggest that galectin-3 is released in response to pulmonary *A*. *fumigatus* infection.

To determine if galectin-3 expression is also induced in humans during *Aspergillus* infection, galectin-3 concentrations were measured in serum samples from patients with invasive pulmonary aspergillosis, chronic pulmonary aspergillosis, an unrelated chronic pulmonary inflammatory disease (sarcoidosis) and healthy individuals. When compared with healthy controls, serum galectin-3 levels were significantly elevated in patients with both forms of pulmonary aspergillosis but not in patients with other pulmonary infections or inflammatory conditions (Fig 1D). Taken as a whole, these data suggest that galectin-3 secretion is induced during *A*. *fumigatus* infection in both mice and humans.

### Galectin-3 deficient mice are more susceptible to *A*. *fumigatus* pulmonary infection

To test the role of galectin-3 in the pathogenesis of *A*. *fumigatus* pulmonary infection, wild-type and galectin-3 deficient mice were infected intratracheally with *A*. *fumigatus* conidia. When compared to wild-type mice, galectin-3 deficient mice exhibited reduced survival when infected (Fig 1E). To determine if the lower survival of galectin-3 deficient mice was a reflection of impaired control of fungal infection, pulmonary fungal burden was measured. Following *A*. *fumigatus* infection, galectin-3 deficient mice exhibited significantly higher pulmonary fungal burden (Fig 1F) and total protein concentrations in the bronchoalveolar lavage (BAL) fluid (Fig 1G) as compared to wild-type animals, suggesting that galectin-3 deficiency was associated with reduced control of fungal growth and increased pulmonary injury [30], respectively. No sex-specific differences in survival and fungal burden were observed in the mice during *A*. *fumigatus* infection (S2A and S2B Fig). Collectively these results suggest that galectin-3 mediates defense against *A*. *fumigatus* infection.

### Galectin-3 binds to the surface of *A*. *fumigatus*, but is not directly fungicidal

**Fig 2:**
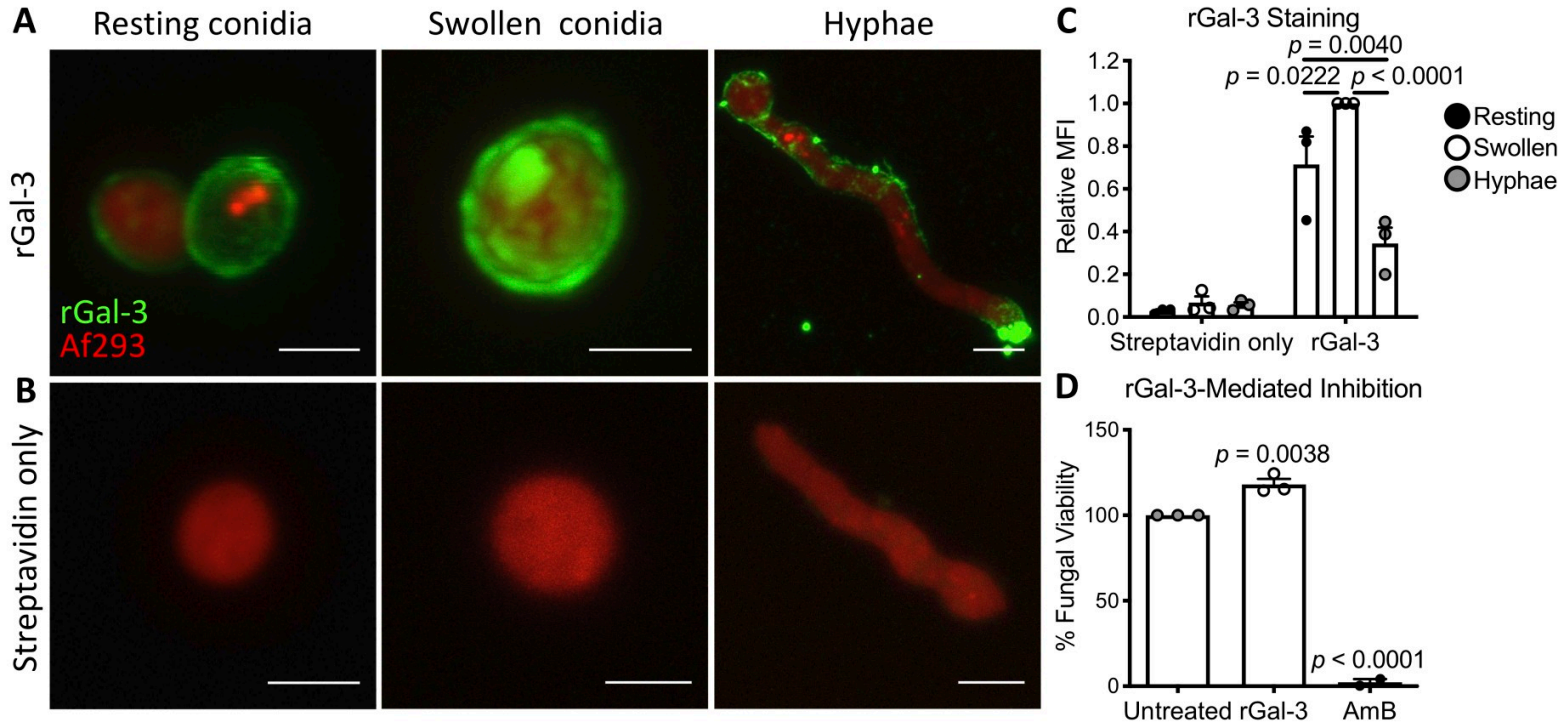
Galectin-3 binds to the surface of *A*. *fumigatus*, but does not affect fungal growth. **(A)** Resting conidia, swollen conidia, and hyphae of red-fluorescent protein-expressing *A*. *fumigatus* strain Af293 (red), stained with biotinylated recombinant galectin-3 followed by fluorescently-labeled streptavidin (rGal-3, green), or **(B)** fluorescently-labeled streptavidin alone (Streptavidin only). Scale bar = 2 μm for resting and swollen conidia, and 5 μm for young hyphae. **(C)** Mean fluorescent intensity (MFI) of (A) and (B), normalized to swollen conidia. Results are the mean of 3 independent experiments. 2-way ANOVA with Sidak’s multiple comparison test. **(D)** The viability of *A*. *fumigatus* Af293 conidia grown for 24 hours in the presence of 850 μg/ml recombinant Galectin-3 (rGal-3) or 8 μg/ml amphotericin B (AmB). Results are the mean of 3 independent experiments. *p* values reported compared to untreated conidia; 1-way ANOVA with Sidak’s multiple comparison test.

As galectin-3 binding to the surface of *C*. *neoformans* and *C*. *albicans* has been reported to mediate direct antifungal effects [11,25], the ability of galectin-3 to bind to, and inhibit the growth of *A*. *fumigatus* was tested. Fluorescent confocal microscopy and flow cytometry studies demonstrated that biotinylated recombinant galectin-3 (rGal-3) bound to the surface of *A*. *fumigatus* resting and swollen conidia, as well as young hyphae (Fig 2A–2C, and S3 Fig). Of the three *A*. *fumigatus* growth stages, galectin-3 bound the strongest to swollen conidia, followed by resting conidia and young hyphae, as determined by measuring the mean fluorescent intensities (MFI) of the stained fungi (Fig 2C, S3B and S3C Fig). Galectin-3 treatment at concentrations as high as 850 μg/ml did not inhibit the growth of *A*. *fumigatus* (Fig 2D), as determined by calcofluor white staining of fungal biomass. These data suggest that galectin-3 is able to bind directly to all morphologies of *A*. *fumigatus*, but does not directly affect fungal growth or viability.

### Galectin-3 deficiency results in reduced neutrophil numbers in the infected airways, leading to impaired control of *A*. *fumigatus* infection

**Fig 3:**
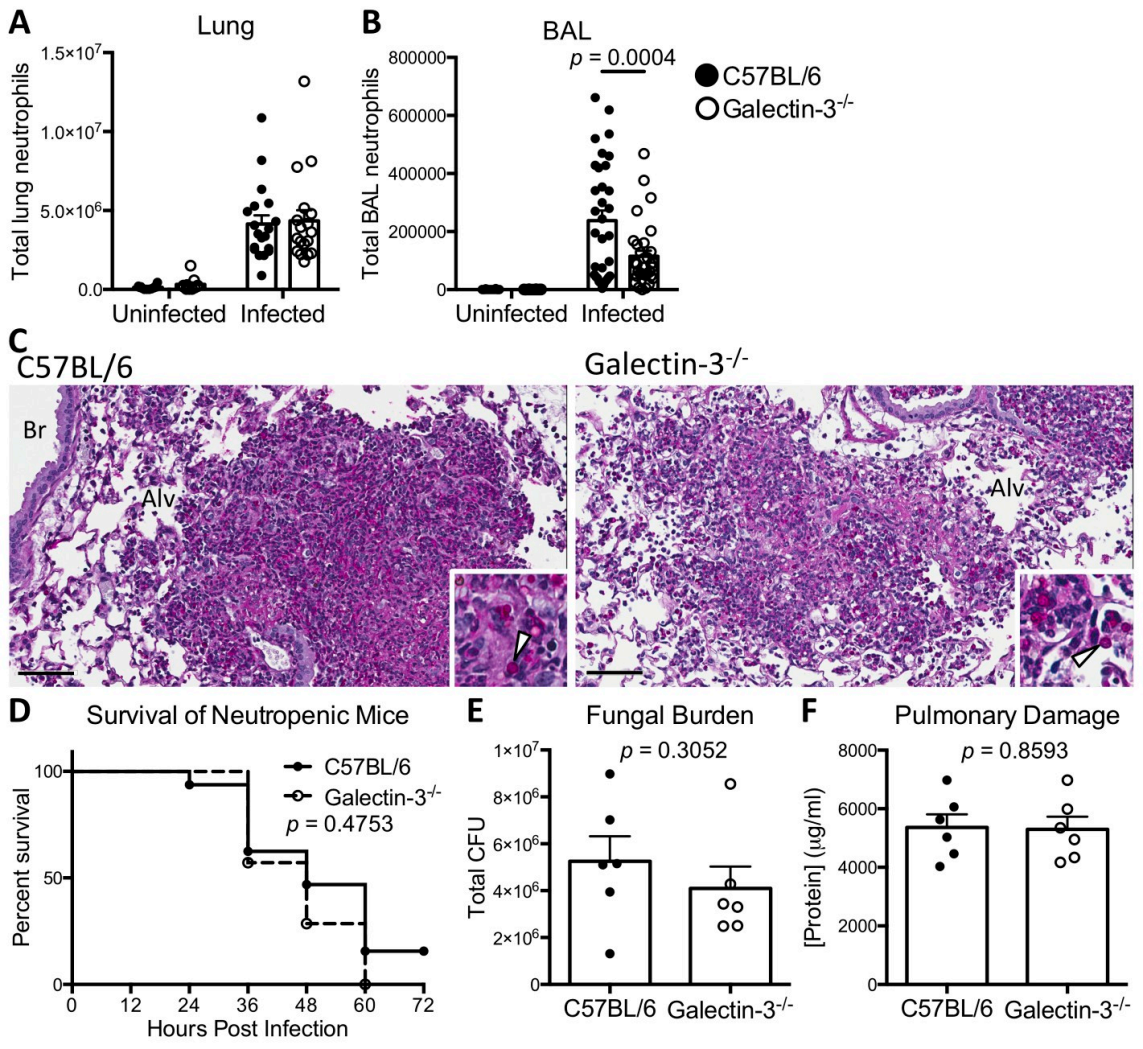
Galectin-3 deficient mice have reduced numbers of neutrophils within the airways, which contributes to impaired control of *A*. *fumigatus* infection. **(A)** Quantification of the neutrophils in total lung tissue and **(B)** BAL fluid of C57BL/6 and galectin-3 deficient mice 36 hours post-infection. For lung tissue, *n* = 12 uninfected and 19 infected C57BL/6 mice; and 11 uninfected and 18 infected galectin-3 deficient mice from 3 independent experiments. For BAL fluid, *n* = 19 uninfected and 33 infected C57BL/6, and 18 uninfected and 32 infected galectin-3 deficient mice from 5 independent experiments. **(C)** Representative periodic acid-Schiff staining of lungs sections from C57BL/6 and galectin-3 deficient mice at 36 hours after infection with Af293 conidia. Inset highlighting conidia (arrows). Alv = alveolar space; Br = bronchiole. Scale bar = 60 μm. **(D)** Survival of neutropenic C57BL/6 and galectin-3 deficient mice infected with Af293 conidia. *n* = 28 C57BL/6 and 23 galectin-3 deficient mice from 2 independent experiments. **(E)** Pulmonary fungal burden and **(F)** total protein content in the BAL fluid of the indicated strains of mice at 36 hours post-infection. *n* = 6 mice per group for (E) and (F). 2-way ANOVA with Sidak’s multiple comparison test for (A) and (B); Mantel-Cox log rank test for (D); unpaired t-test for (E) and (F).

To characterize the role of galectin-3 in the immune response towards *A*. *fumigatus* infection, the immune cell populations in the whole lung digest and BAL fluid of wild-type and galectin-3 deficient mice were analyzed. No differences between wild-type and galectin-3 deficient mice were observed in the number of neutrophils, alveolar macrophages, eosinophils, and inflammatory monocytes present in total lung samples (Fig 3A and S4A Fig). However, significantly fewer neutrophils and inflammatory monocytes were recovered from the BAL fluid of infected galectin-3 deficient animals as compared to infected wild-type controls (Fig 3B and S4B Fig). Consistent with these data, examination of pulmonary tissue sections revealed that fungal lesions in galectin-3 deficient animals were surrounded by reduced levels of inflammation and leukocyte numbers as compared to wild-type mice (Fig 3C).

The numbers of both neutrophils and inflammatory monocytes were reduced in the airways of galectin-3 deficient mice during *A*. *fumigatus* infection. As neutrophils play a critical role in the innate defense against *A*. *fumigatus* infection [2], we hypothesized that galectin-3-mediated neutrophil migration to the site of infection is the primary mechanism by which this lectin contributes to host resistance against this organism. To test this hypothesis, we next determined the effects of neutrophil depletion on the susceptibility of wild-type and galectin-3 deficient mice to *A*. *fumigatus* challenge [31]. Neutrophil depletion abrogated the differences in susceptibility to *A*. *fumigatus* between wild-type and galectin-3 deficient mice. No statistical differences in survival (Fig 3D), pulmonary fungal burden (Fig 3E) or pulmonary damage (Fig 3F) were observed between neutrophil-depleted wild-type and galectin-3 deficient mice. These data suggest that the differences in neutrophil recruitment to the airspaces previously observed between immunocompetent wild-type and galectin-3 deficient mice likely plays a key role in the increased susceptibility of galectin-3 deficient mice to *A*. *fumigatus* pulmonary infection.

### Galectin-3 is required for optimal neutrophil migration out of the vasculature and into the airways during *A*. *fumigatus* infection

**Fig 4:**
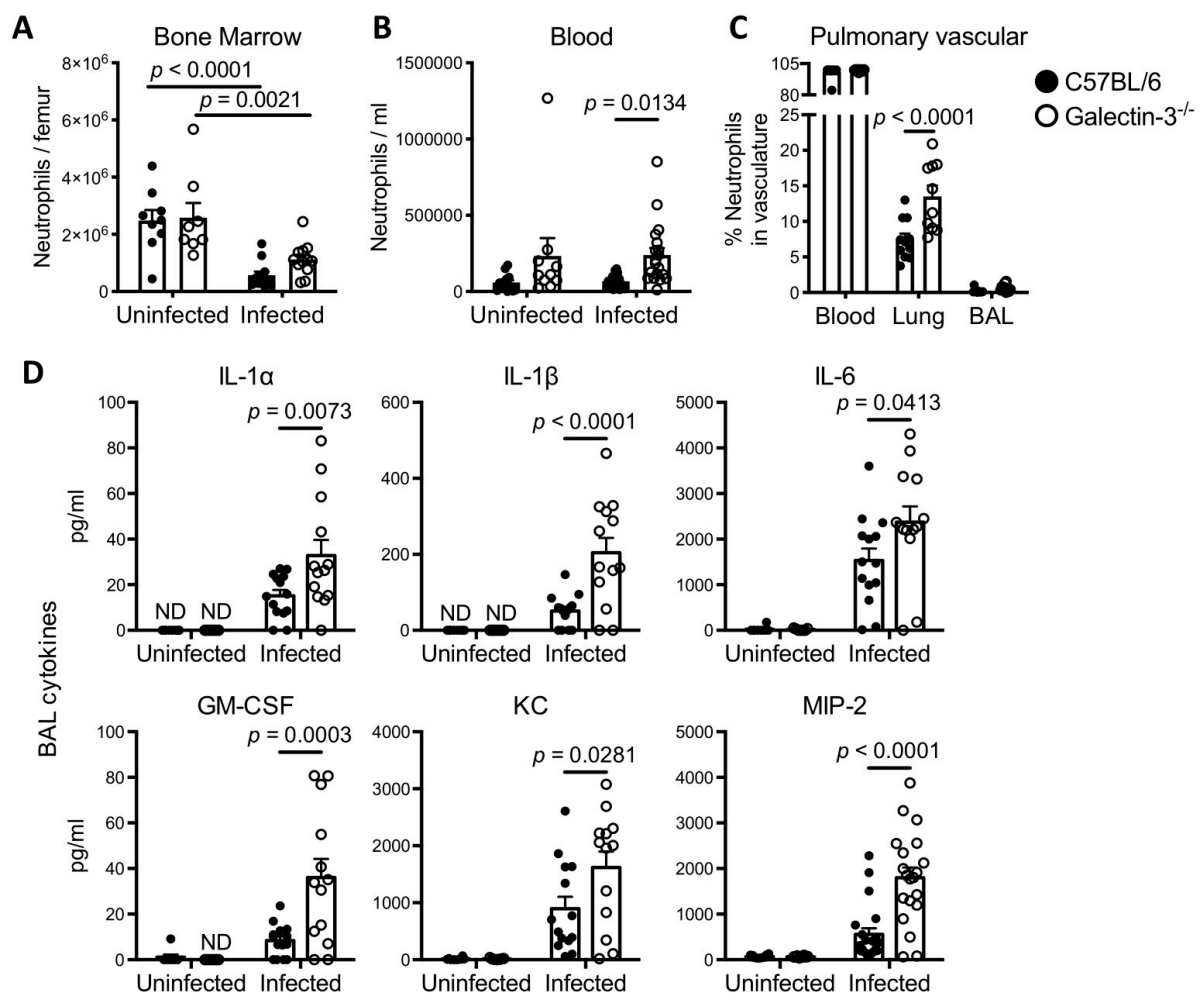
During pulmonary *A*. *fumigatus* infection, galectin-3 deficient mice have reduced neutrophil migration to the airways despite higher levels of pulmonary pro-inflammatory and chemotactic cytokines. **(A)** Quantification of the total bone marrow neutrophils and **(B)** neutrophils in the blood of C57BL/6 and galectin-3 deficient mice 36 hours post-infection. For bone marrow quantification, *n* = 9 uninfected and 12 infected C57BL/6, and 8 uninfected and 12 infected galectin-3 deficient mice from 2 independent experiments. For blood quantification, *n* = 13 uninfected and 19 infected C57BL/6, and 10 uninfected and 19 infected galectin-3 deficient mice from 3 independent experiments. **(C)** Percentage of pulmonary neutrophils associated with the vasculature of infected C57BL/6 and galectin-3 deficient mice as detected by intravascular staining and flow cytometry, 36 hours post-infection. *n* = 11 C57BL/6 and 10 galectin-3 deficient mice from 2 independent experiments. **(D)** Concentrations of IL-1α, IL-1β, IL-6, GM-CSF, KC, and MIP-2 as measured in the BAL fluid of mice 36 hours post infection. For IL-1α, IL-1β, IL-6, GM-CSF and KC, *n* = 8 uninfected and 14 infected C57BL/6, and 7 uninfected and 13 infected galectin-3 deficient mice from 2 independent experiments. For MIP-2, *n* = 12 uninfected and 21 infected C57BL/6, and 10 uninfected and 20 infected galectin-3 deficient mice from 3 independent experiments. ND: non-detected. ns: not significant; 2-way ANOVA with Sidak’s multiple comparison test.

To determine whether the reduced numbers of neutrophils within the airways of galectin-3 deficient mice were due to impaired neutrophil production, we next determined the total neutrophil numbers in the bone marrow and peripheral blood. No differences in the number of bone marrow neutrophils were observed between *A*. *fumigatus* infected wild-type and galectin-3 deficient mice (Fig 4A). Both strains of mice exhibited a significant reduction of neutrophils in the bone marrow following infection, possibly reflecting mobilization of bone marrow neutrophil pools to the site of infection. In contrast, higher numbers of neutrophils were found in the blood of galectin-3 deficient mice, suggesting the possibility that they were unable to reach the airways during infection (Fig 4B). To determine the distribution of neutrophils between the pulmonary vasculature and lung parenchyma of galectin-3 deficient mice during infection, intravascular neutrophil staining was performed [32,33]. Consistent with the results of neutrophil quantification in the blood and BAL fluid, a higher proportion of pulmonary neutrophils were found within the vasculature of galectin-3 deficient mice as compared to wild-type mice (Fig 4C).

To test if the impaired neutrophil recruitment to the airways seen in galectin-3 mice was a consequence of reduced production of chemotactic factors, we determined the levels of cytokines in BAL fluid in wild-type and galectin-3 deficient mice at 36 hours post infection. To our surprise the levels of multiple cytokines, including IL-1α, IL-1β, IL-6, granulocyte macrophage colony-stimulating factor (GM-CSF), keratinocyte-derived chemokine (KC), and macrophage inflammatory protein-2 (MIP-2), which are involved in the recruitment or activation of neutrophils and other inflammatory cells were higher in the BAL fluid of galectin-3 deficient mice compared to wild-type mice after *A*. *fumigatus* infection (Fig 4D). In contrast, the levels of numerous cytokines that have been reported to be produced by neutrophils [34–38] were found to be lower in the BAL fluid of galectin-3 deficient mice than in wild-type mice, including IL-1 receptor antagonist (IL-1Ra), IL-12, IL-17, monocyte chemoattractant protein 1 (MCP-1) and “regulated upon activation normal T cell expressed and secreted” (RANTES) (S5 Fig). These data suggest that the reduced numbers of airway neutrophils in galectin-3 deficient mice is not a consequence of reduced production of chemotactic factors. Furthermore, the lower BAL levels of cytokines that have been reported to be produced by neutrophils are consistent with the histopathology and flow cytometry findings of reduced neutrophil numbers in the airspaces of galectin-3 deficient mice during *A*. *fumigatus* infection.

Taken together, these data suggest that galectin-3 plays an important role in enhancing immune cell migration from the pulmonary vasculature to the site of infection within the pulmonary airspaces that is independent of bone marrow granulopoiesis, and pulmonary chemokine production.

### Galectin-3 deficient neutrophils are not impaired in their ability to kill *A*. *fumigatus*

**Fig 5:**
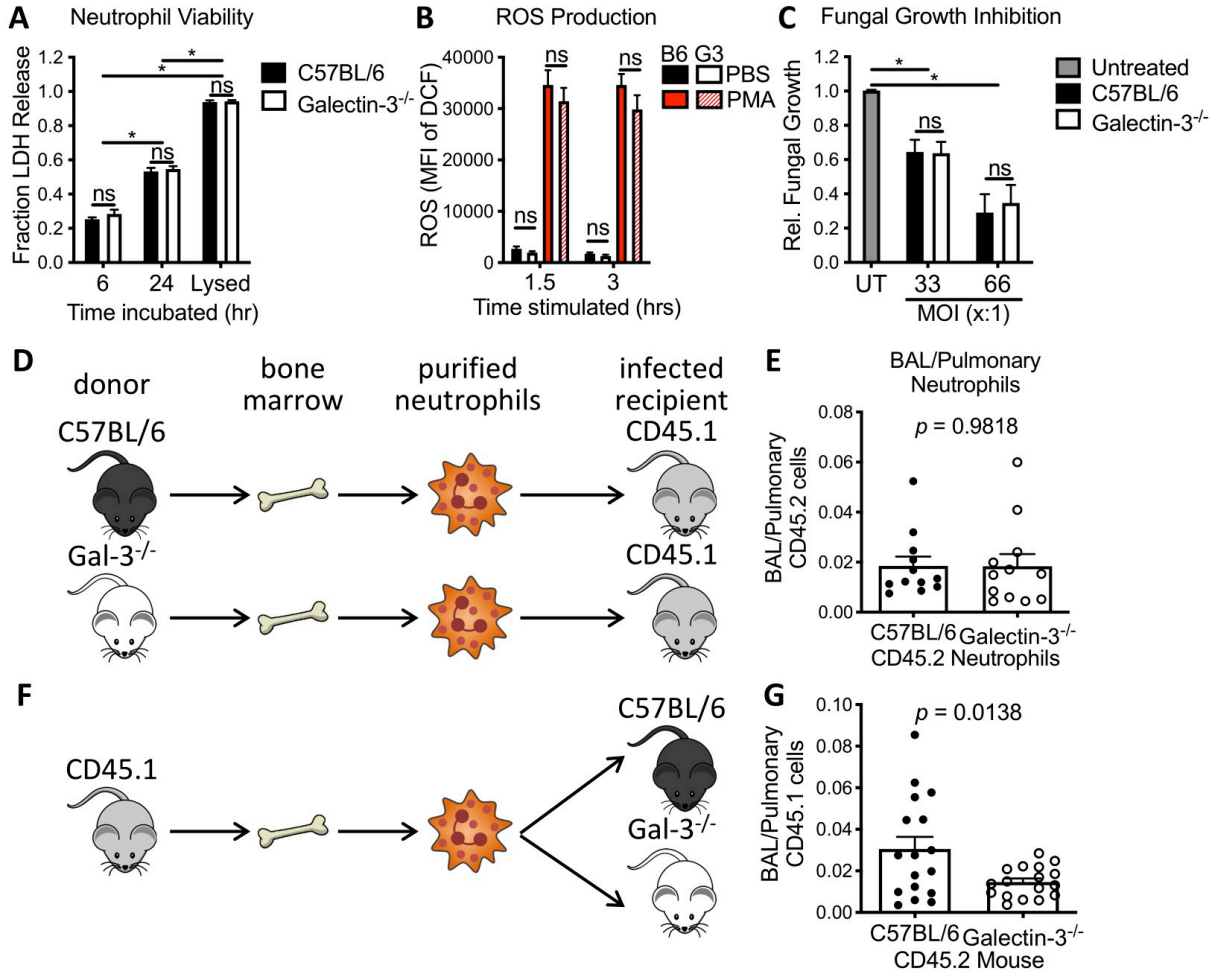
Neutrophil-intrinsic galectin-3 is dispensable for neutrophil antifungal activity *in vitro* and for neutrophil migration during pulmonary *A*. *fumigatus* infection. **(A)** The viability of murine bone-marrow-isolated neutrophils (BMNs) as determined by lactose dehydrogenase (LDH) release at indicated time points. *n* = 3 and 4 independent experiments for 6 and 24 hrs, respectively. **(B)** The ability of C57BL/6 (B6) and galectin-3 deficient (G3) BMNs to produce reactive oxygen species (ROS) in response to phorbol myristate acetate (PMA) stimulation, as determined by CM-H_2_DCFDA (DCF) fluorescence measured at indicated time points. *n* = 4 independent experiments. **(C)** Antifungal activity of BMNs from the indicated mouse strains against Af293 hyphae over 18 hrs, relative to growth of fungi not treated with neutrophils (untreated, UT). *n* = 5 and 9 independent experiments for MOIs of 33:1 and 66:1, respectively. **(D)** Experimental design for adoptive transfer of neutrophils from the bone marrow of CD45.2 C57BL/6 and galectin-3 deficient mice into infected B6-CD45.1 mice. **(E)** The fraction of total pulmonary CD45.2 neutrophils that were found in the BAL. *n* = 12 mice per group from 2 independent experiments. **(F)** Experimental design for adoptive transfer of neutrophils were isolated from the bone marrow of B6-CD45.1 mice into infected C57BL/6 and galectin-3 deficient mice. **(G)** The fraction of total pulmonary CD45.1 neutrophils that were found in the BAL. *n* = 17 mice per group from 4 independent experiments. ns: not significant. *: *p* < 0.05; 2-way ANOVA with Sidak’s multiple comparison test for (A—C); unpaired t-test for (E) and (G).

To determine whether the increased susceptibility of galectin-3 deficient mice is due to reduced numbers of neutrophils or impaired antifungal immunity of neutrophils, we next tested the role of galectin-3 in neutrophil function. To test this hypothesis the viability, reactive oxygen species (ROS) production and antifungal activity of neutrophils purified from the bone marrow of both wild-type and galectin-3 deficient mice were evaluated *in vitro*. Wild-type and galectin-3 deficient neutrophils exhibited similar loss of viability over 24 hours as determined by lactose dehydrogenase (LDH) release at 6 and 24 hours of culture (Fig 5A). Neutrophils from both strains of mice also generated similar levels of ROS following 1.5 hours of stimulation with phorbol myristate acetate (PMA) (Fig 5B). Finally, galectin-3 deficient and wild-type neutrophils purified from bone marrow were able to inhibit the growth of young *A*. *fumigatus* hyphae to the same extent (Fig 5C), suggesting that galectin-3 is not required for normal neutrophil antifungal activity. Collectively, these data suggest that neutrophils from galectin-3 deficient mice do not exhibit a global defect in function.

### Adoptive transfer of neutrophils reveals that neutrophil-intrinsic galectin-3 is dispensable for neutrophil migration within the lungs

Given the normal *in vitro* function of galectin-3 deficient neutrophils, an adoptive transfer approach was taken to test the hypothesis that extracellular galectin-3, rather than neutrophil-intrinsic galectin-3, is required for normal neutrophil migration during pulmonary *A*. *fumigatus* infection.

To confirm that neutrophil-intrinsic galectin-3 was dispensable for migration to the airways, neutrophils were purified from the bone marrow of both wild-type and galectin-3 deficient naïve mice (both of which express CD45.2), and transferred to infected wild-type B6-CD45.1 mice via tail vein injection 24 hours post *A*. *fumigatus* infection (Fig 5D). At 12 hours post adoptive transfer, no differences in the fraction of pulmonary neutrophils recruited to the airways were observed between wild-type and galectin-3 deficient donor neutrophils, suggesting that neutrophil-intrinsic galectin-3 is dispensable for efficient neutrophil recruitment to the airways (Fig 5E).

To confirm that the neutrophils require extrinsic galectin-3 for normal migration to the airways, the inverse experiment was conducted whereby neutrophils were purified from the bone marrow of B6-CD45.1 mice and administered intravenously to both wild-type and galectin-3 deficient mice following *A*. *fumigatus* infection (Fig 5F). A significantly greater proportion of the pulmonary B6-CD45.1 neutrophils were found in the airways of recipient wild-type mice as compared to the galectin-3 deficient animals (Fig 5G), suggesting that galectin-3 from lung cells other than neutrophils are necessary for effective neutrophil recruitment to the airways.

### Neutrophils exhibit galectin-3-dependent motility and displacement towards *A*. *fumigatus* in infected mouse lungs

**Fig 6:**
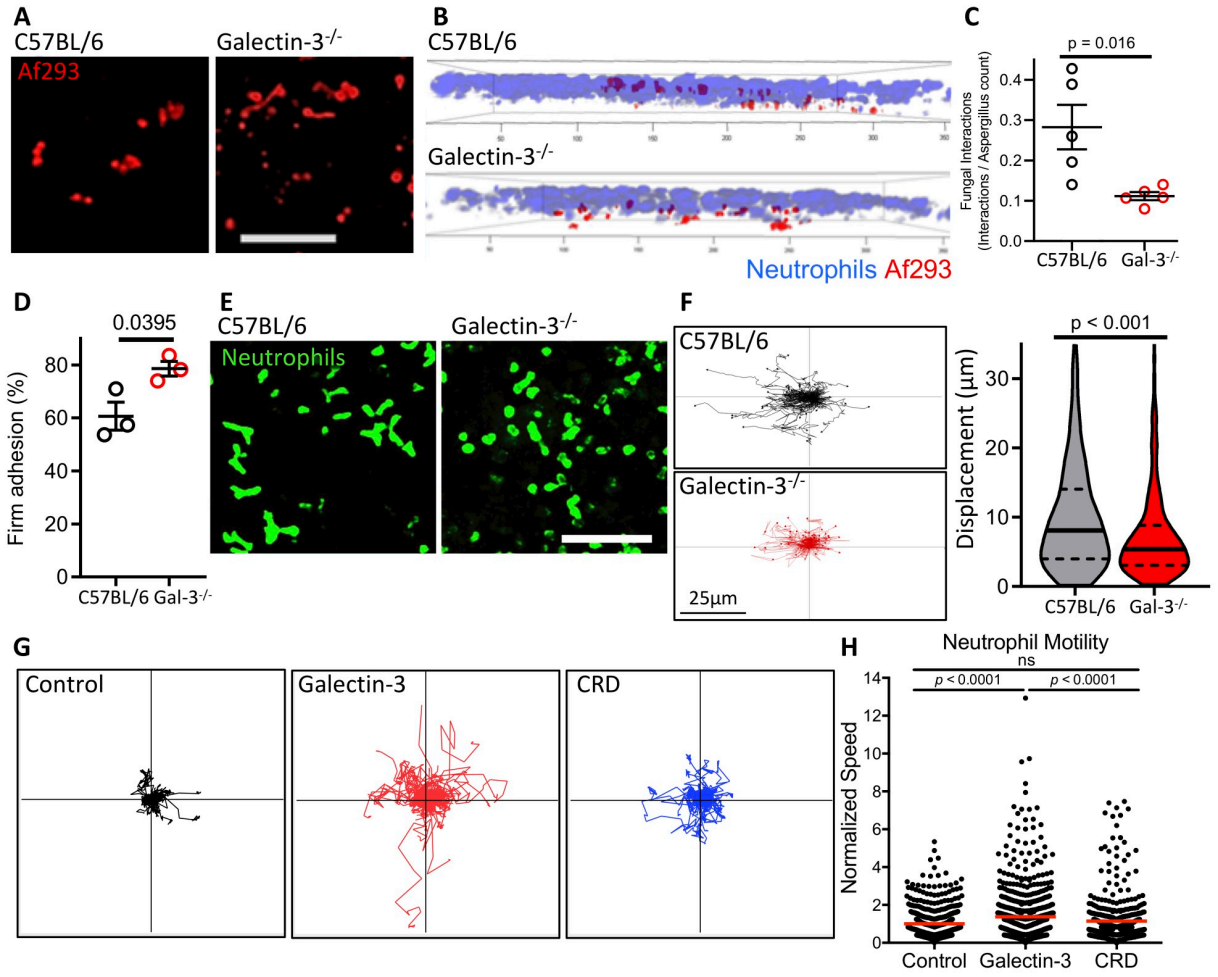
Galectin-3 enhances mouse and human neutrophil motility. **(A)** Intravital imaging revealed that galectin-3 deficient lungs had more *A*. *fumigatus* (red) germination and growth at 16 hrs post-infection, suggesting reduced control of the fungal infection. **(B)** Both neutrophils and *A*. *fumigatus* were more closely associated with the same focal plane in C57BL/6 than galectin-3 deficient lungs, and **(C)** had more instances of co-localization. 5 mice per group. **(D)** A higher proportion of neutrophils were firmly adhered in galectin-3 deficient lungs. 3 mice per group. Student’s t-test for (C) and (D). **(E)** A higher degree of polarization of the neutrophils was observed in C57BL/6 lungs, compared to galectin-3 deficient lungs, which remain rounded. **(F)** Movements of mouse neutrophils tracked *in vivo* show reduced motility of neutrophils in galectin-3 deficient lungs. 3 mice per group, Mann-Whitney non-parametric test. **(G)** Human peripheral blood neutrophils were incubated in the presence of 1 μM recombinant full-length galectin-3 or the carbohydrate-recognition domain of galectin-3 (CRD) and their movements tracked by live-cell imaging. **(H)** The average speed of each live cell was then normalized to the average speed in the absence of galectin-3 (control). Data pooled from 3 independent experiments. ns: not significant; Kruskill-Wallis test with Dunn’s multiple comparison test.

To further probe the role of galectin-3 in the migration of the neutrophils to the airways, the activity of the neutrophils was monitored in the lungs of infected mice using intravital imaging. Consistent with our finding of increased pulmonary fungal burden in galectin-3 deficient mice (Fig 1F), more germinated *A*. *fumigatus* and hyphae were observed in the lungs of these animals (Fig 6A). Within the lungs of galectin-3 deficient mice, fewer neutrophils were found to occupy the same focal plane as *A*. *fumigatus* as compared to those in wild-type lungs (Fig 6B). Similarly, lower rates of neutrophil-fungal co-localization were observed in galectin-3 deficient mice (Fig 6C and S1 Movie). A significantly higher proportion of the neutrophils in the galectin-3 deficient lungs exhibited firm adherence as compared to wild-type mice (Fig 6D and S1 Movie), suggesting that neutrophil adhesion to pulmonary endothelial cells was not impaired. Consistent with these findings, flow cytometry analysis of pulmonary endothelial cells from infected galectin-3 deficient mice revealed normal or increased levels of leukocyte adhesion molecule expression (S6 Fig). However, neutrophils within the lungs of galectin-3 deficient mice exhibited a predominately round morphology, as well as reduced non-directional movement and overall displacement as compared with wild-type neutrophils (Fig 6E and 6F). These findings suggest that galectin-3 may play a role in enhancing neutrophil motility, allowing for the neutrophils to efficiently exit the blood stream and interact with *A*. *fumigatus* within the airways.

### Extracellular galectin-3 enhances neutrophil motility

The results of the adoptive transfer experiments, intravital studies, and the high levels of soluble galectin-3 found in the serum of mice and humans with *Aspergillus* infections suggest that extracellular galectin-3 directly enhances neutrophil motility and migration during pulmonary *Aspergillus* infection. To test this hypothesis, the effects of recombinant galectin-3 on human neutrophil motility were examined using live cell imaging. Human peripheral blood neutrophils were treated with either full-length galectin-3 or a truncated construct of galectin-3 containing only the CRD, and neutrophil motility was quantified using single cell tracking analysis. Treatment of human neutrophils with full-length galectin-3 exposure significantly enhanced motility on collagen-coated surfaces, resulting in migration paths that were significantly longer in full-length galectin-3 treated samples (Fig 6G and 6H). Neutrophils treated with the galectin-3 CRD alone exhibited similar motility to the untreated control cells, suggesting that multimerization of galectin-3 is required to enhance neutrophil motility.

## Discussion

**Fig 7:**
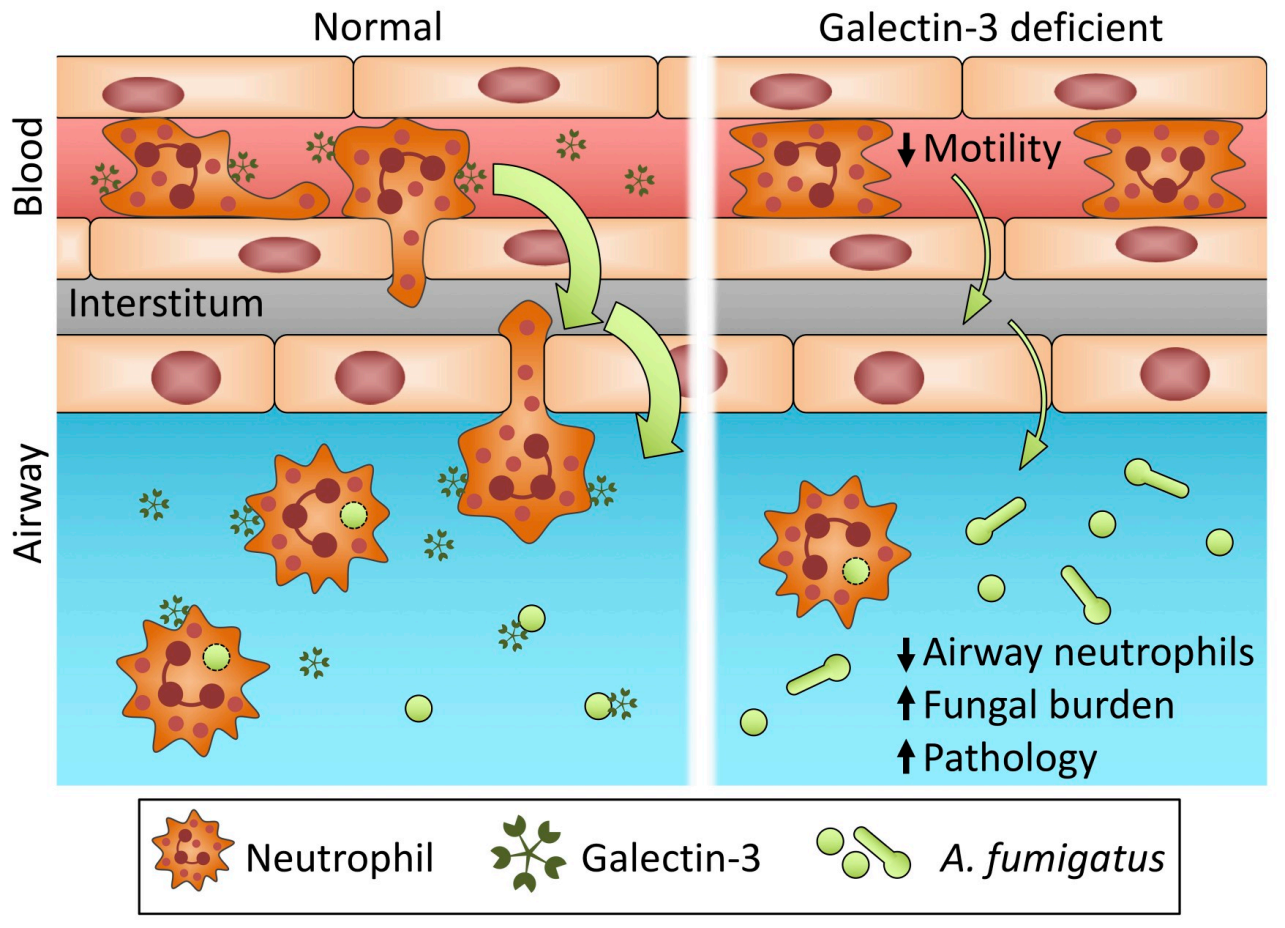
Graphical abstract depicting the role of extracellular galectin-3 in enhancing neutrophil motility, resulting in increased migration to the airways and greater control of the infection during pulmonary aspergillosis.

Our findings identify a novel role for galectin-3 in the host defense against *A*. *fumigatus* pulmonary infection. Galectin-3 levels were significantly increased during both mouse and human *A*. *fumigatus* infection. Surprisingly, in contrast to previous studies of the role of galectin-3 in host resistance to pathogenic yeast, galectin-3 exhibited no direct antifungal activity against the mold *A*. *fumigatus*. In mice infected with *A*. *fumigatus*, galectin-3 mediated the efficient egress of neutrophils from the bloodstream to the site of infection. Neutrophil adoptive transfer experiments and intravital microscopy revealed that stromal, rather than neutrophil-intrinsic galectin-3 directly enhanced neutrophil motility during *A*. *fumigatus* infection. A role for extracellular galectin-3 in enhancing neutrophil motility was confirmed with studies of galectin-3 treatment of human neutrophils *in vitro* (Fig 7). These studies reveal a novel mechanism of action for this host lectin in pulmonary fungal infections.

Through our immunophenotyping and neutrophil adoptive transfer studies we found that extrinsic galectin-3 is required for effective neutrophil egress from the blood vessels and into the site of the infection within the airways. In models of bacterial [6,7,39] and parasitic infection [8], we and others have reported that galectin-3 plays a role in neutrophil recruitment to the site of infection, although the mechanisms underlying this observation are poorly understood [6]. Early studies demonstrated that galectin-3 was not directly chemotactic, but could bind directly to neutrophils and induce their activation [6,40,41]. More recently, intravital imaging of neutrophil interactions with cremasteric postcapillary venules following TNF-α treatment demonstrated a role for neutrophil-intrinsic galectin-3 in early endothelial cell adherence. In these studies, neutrophil-intrinsic galectin-3 was required for E-selectin-dependent tethering and rolling of neutrophils [42]. These observations differ from our findings during *A*. *fumigatus* pulmonary infection, in which neutrophil-intrinsic galectin-3 was dispensable for neutrophil recruitment, and galectin-3 deficient mice exhibited increased firm adherence of neutrophils to endothelial cells. These seemingly contrasting findings likely reflect differences in neutrophil migration from cremasteric venules versus pulmonary capillaries. Alveolar capillaries are smaller in diameter than neutrophils, which must deform themselves to pass through this smaller opening and slowing their velocity inside the blood vessel [43]. As a consequence, neutrophil rolling and tethering are not thought to occur in pulmonary capillaries [44]. Thus, while neutrophil-intrinsic galectin-3 may play an important role in early neutrophil adhesion to endothelial cells in larger vessels, our studies reveal that extracellular galectin-3 acts to stimulate neutrophil motility and crawling. While galectin-3 binding to the surface of neutrophils was not studied, our findings and those of the studies mentioned above suggest that this likely occurs.

While our study focused on the effects of galectin-3 on neutrophils, we also observed reduced numbers of monocytes in the airways of galectin-3 deficient mice during *A*. *fumigatus* infection. Monocytes also play an important role in host resistance to *A*. *fumigatus* pulmonary aspergillosis [45]. Following selective depletion of inflammatory monocytes, it was reported that while neutrophils were able to interact with and phagocytose *A*. *fumigatus* conidia, they were unable to efficiently kill the fungus. It is therefore possible that galectin-3 mediated monocyte migration may also contribute to host resistance to *A*. *fumigatus*, although further investigation is required to confirm this hypothesis.

The findings of this study identify a novel role for extracellular galectin-3 in host defense against *A*. *fumigatus* infection through enhancing neutrophil motility and egress from the pulmonary vasculature. Further studies will be required to elucidate the neutrophil receptors and signalling pathways involved in this process.

## Materials and Methods

### Study design

At least 3 independent experiments were conducted for *in vitro* studies. For experiments involving mice, 4–8 mice were used per group per experiment and experiments were replicated as indicated. Samples were randomly allocated to the different groups. Investigators were blinded during the survival experiments. No data were excluded from analysis.

### Strains and reagents

*Aspergillus fumigatus* strain Af293 was generously provided by Dr. P. Magee. For experiments, conidia were cultivated on Yeast-Peptone-Dextrose (YPD) agar plates for 6 days at 37°C, at which point the conidia were harvested by gentle washing of the mycelial mat with phosphate-buffered saline (PBS) + 0.05% (v/v) Tween-20 (PBS-T). Galectin-3 deficient (B6.Cg-*Lgals3*^*tm1Poi*^/J) mice were purchased from The Jackson Laboratory and bred at our facility, and wild-type controls (C57BL/6) and C57BL/6-CD45.1 mice were purchased from The Jackson Laboratory and Charles River Laboratories. The anti-galectin-3 antibody was purified from the M3/38 hybridoma cell line using GE Healthcare HiTrap Protein G HP column as previously published [46]. Murine bone marrow, blood, lung digestate, and BAL fluid were stained for flow cytometry using fluorescently-labeled antibodies detailed in S1 Table. Recombinant galectin-3 was produced in *Escherichia coli* as previously described [46]. Purified galectin-3 was passed through an ActiClean Etox endotoxin-removing column (Sterogene) for *in vitro* experiments, ensuring that the endotoxin level is <1 pg/μg of protein [8]. The CRD construct of galectin-3 lacking the *N-*terminal domain required for oligomerization was produced as previously described [40]. Biotinylation of galectin-3 was prepared as previously described by using EZ-link Sulfo-NHS-LC-Biotin (PIERCE) as previously described [47].

### Ethics Statements

All experiments involving mice were approved by the Animal Care Committees of the McGill University Health Centre or the University of Calgary. Clinical studies using patient samples were approved by the Nagasaki University School of Medicine Research Ethics Committee (Approval number: 17091125–5) and the University of Florida Institutional Review Board (protocol: 073–2008). Subjects provided written, informed consent prior to participation in the study. Peripheral blood was collected from healthy volunteers under a license from “Comité d’éthique de la recherche du CHU de Québec-Université Laval”.

### Endotracheal conidia infection

Mice 8–10 weeks of age were anaesthetized with isoflurane and endotracheally infected with 5 × 10^7^ Af293 conidia in 50 μl PBS-T. Mice were monitored daily for signs of distress and moribund animals were euthanized by CO_2_ overdose. To induce neutropenia, mice received an intraperitoneal injection of 200 μg of anti-Ly6G antibody (clone 1A8, BioXcell) starting 24 hours prior to infection, and every 48 hours until the end of the experiment. Neutrophil depletion was confirmed via differential cell counts of peripheral blood samples.

### Histopathological preparation and staining

At 36 hours post infection, mice were euthanized, their lungs removed, inflated with 10% PBS-buffered formalin, and fixed in formalin. Fixed lungs were embedded in paraffin, and 4 μm-thick sections were stained with either immunohistochemical staining using the anti-galectin-3 antibody and visualized by staining with donkey-anti-rat antibody conjugated to horseradish peroxidase and developed using 3,3'-diaminobenzidine, or periodic acid Schiff (PAS) stain.

### Quantification of galectin-3 in serum

Following euthanasia, cardiac puncture was performed on the mice and blood collected in Microvette 500 Z-Gel tubes (Sarstedt) and centrifuged to yield serum.

Human serum samples were collected from patients who were diagnosed with pulmonary aspergillosis in Nagasaki University Hospital, Japan. Control samples were obtained from healthy volunteers, and patients with pulmonary sarcoidosis who had no signs of fungal infection and were not receiving corticosteroids.

Additional human sample collection was conducted by the Aspergillus Technology Consortium (AsTeC). Blood was collected in sterile vacuum tubes with no additives by venipuncture or from central catheter, if present. The serum fraction was separated from cells by centrifugation. No further processing was performed and aliquots were prepared using aseptic techniques. All samples and clinical data were de-identified and linked to an anonymous study ID number at the AsTeC Biorepository.

All samples were stored at -80°C until use. Galectin-3 was quantified in mouse serum samples using the Mouse Galectin-3 DuoSet ELISA (R&D systems, DY1197) according to manufacturer’s instructions. Galectin-3 levels were measured in the human samples by the Human Galectin-3 Quantikine ELISA kit (R&D systems), as per manufacturer’s instructions.

### Pulmonary fungal burden determination

Excised lungs were resuspended in PBS and homogenized, and the resulting homogenate was diluted and plated onto YPD agar containing 1% (v/v) penicillin/streptomycin via sterile glass beads. Colonies were counted following an overnight incubation at 37°C.

### Measurement of proteins released into the BAL fluid

Following euthanasia, mice were tracheotomised and a blunt 18-gauge needle was inserted into the trachea. Lungs were then lavaged with 1 ml PBS, and the resulting BAL fluid was centrifuged at 100 *xg* for 10 mins and the supernatant collected for total protein and cytokine analyses. Total protein content of the BAL fluid was determined using the bicinchoninic acid (BCA) assay (Thermo Scientific), as per manufacturer’s instructions.

Cytokine analysis of the BAL fluid was performed using a custom mouse cytokine multiplex array (IL-1α, IL-1β, IL-6, IL-12p70, IL-17, GM-CSF, KC, MCP-1, and RANTES; Quansys Biosciences), as per manufacturer’s instructions. Individual ELISAs were also used to quantify IL-1α (eBioscience), KC, MIP-2, and IL-1Ra (all from R&D Biosystems), as per manufacturer’s instructions.

### Immunofluorescent *A*. *fumigatus* staining by recombinant galectin-3

*A*. *fumigatus* Af293 expressing the red fluorescent protein (RFP) mRFP1 [48] was grown in Dulbecco’s Modified Eagle Medium (DMEM). Samples were washed with PBS and blocked with PBS containing 3% (w/v) 1x crystallized BSA (blocking buffer). Samples were washed and incubated with blocking buffer containing 75 μg/ml biotinylated recombinant galectin-3. Samples were washed and incubated with blocking buffer containing 2.5 μg/ml streptavidin conjugated to Alexa Fluor 488 (Jackson Immunoresearch), and fixed with 4% (w/v) paraformaldehyde (PFA) in PBS. Coverslips were then mounted in SlowFade Diamond Antifade mounting medium (Thermo) and sealed.

Samples were acquired on an Olympus confocal fluorescent microscope using a 63x/1.40 Oil objective lens at a resolution of 1024X1024, using 488 and 543 nm lasers to excite Alexa Fluor 488 and RFP, respectively. The channels were acquired separately, with Alexa Fluor 488 being detected at 492–545 nm, and RFP at 567–754 nm. Z-stacks were acquired with a spacing of 0.45 μm. Stacks were combined using the “Maximum Intensity Projection” algorithm of the ImageJ software and exported as a.jpeg image.

For analysis of rGal-3 binding to conidia by flow cytometry, conidia were grown for 4 hrs in Brian media to yield swollen conidia. Conidia were then stained as above and data were acquired on an LSR Fortessa flow cytometer using FACSDiva software (BD Biosciences). Following acquisition, data were analyzed using FlowJo software version 10 (FlowJo, LLC).

### Direct fungal growth inhibition by recombinant galectin-3

*A*. *fumigatus* Af293 conidia were grown for 24 hours in DMEM supplemented with 850 μg/ml recombinant galectin-3 or 8 μg/ml amphotericin B. The resulting biomass was then stained with 1 mg/ml solution of calcofluor white, and the resulting fluorescence measured with an excitation of 340 nm and an emission at 440 nm.

### Cellular recruitment analysis by flow cytometry

Femurs were harvested and bone marrow collected via centrifugation. Blood was harvested by cardiac puncture and collected in BD Vacutainer vials coated with Lithium Heparin. Lungs were lavaged twice with 1 ml PBS, and the resulting BAL fluid was centrifuged at 100 *xg* for 10 mins. The resulting cell pellets recovered after centrifugation were combined. Lungs were minced with scalpel blades and digested by 150 units/ml collagenase type IV (Sigma) in Roswell Park Memorial Institute Medium (RPMI) supplemented with 5% (v/v) Fetal Bovine Serum (FBS). Samples were dispersed through an 18G needle, and strained through a 70 μm cell strainer.

Erythrocytes were lysed by incubating the samples with ACK buffer, and the remaining cells were stained with 0.1% (v/v) fixable viability dye (eBioscience). Samples were washed in PBS + 2% (v/v) FBS (staining buffer), and incubated with unlabelled anti-CD16/32 antibodies (FcBlock; BD Pharmingen) to block the Fc receptors. Samples were stained *ex vivo* with the following fluorescently-labelled antibodies: CD45-APC-Cy7, CD11b-PE-CF594, CD11c-APC, Ly6G-PE, Ly6C-Alexa Fluor 700, and SiglecF-brilliant violet 421. Cells were washed with staining buffer and fixed with 2% (w/v) PFA in PBS. For intracellular galectin-3 staining, extracellular staining was modified by substituting Ly6G-PE for Ly6G-brilliant violet 650 and adding CD31-Alexa Fluor 488. Samples were then permeabilized with BD Cytofix/Cytoperm and stained with galectin-3-PE antibody. All samples were subsequently washed and resuspended in PBS prior to data acquisition. Samples were spiked with BD CountBright absolute counting beads and data was acquired on an LSR Fortessa flow cytometer using FACSDiva software (BD Biosciences). Following acquisition, data were analyzed by using FlowJo software version 10 (FlowJo, LLC), with cell subsets being defined as follows: neutrophils, CD45^+^ Ly6G^+^ CD11c^−^ CD11b^+^; alveolar macrophages, CD45^+^ CD11c^+^ siglecF^+^ CD11b^neg/low^; eosinophils, CD45^+^ CD11b^+^ CD11c^−^ siglecF^+^; inflammatory monocytes, CD45^+^ Ly6C^+^ Ly6G^−^ CD11b^+^. For gating strategy, see S7 Fig.

### Staining of vasculature-associated cells

To determine the proportion of cells in the lung digest that were of intravascular origin, *in vivo* intravascular staining with anti-CD45 antibody was performed [32]. Three minutes prior to euthanasia, mice were injected intravenously with 300 μl PBS containing 3 μg anti-CD45-PE-CF594 (clone 30-F11). Following euthanasia blood, BAL fluid and lungs were collected and processed as above. Samples were stained *ex vivo* as above with the following antibodies: CD45-APC-Cy7, CD11b-FITC, CD11c-APC, Ly6G-PE, Ly6C-Alexa Fluor 700, and SiglecF-brilliant violet 421. Samples were analyzed by flow cytometry, with neutrophil populations identified as defined as above. Cells associated with the vasculature were defined as being positive for both CD45 markers. For gating strategy, see S8 Fig.

### Mouse bone marrow neutrophil isolation

Bone marrow was collected from the femurs and tibias of mice, 8–10 weeks of age. Neutrophils were isolated using the mouse MACS Neutrophil Isolation Kit and LS columns (Miltenyi Biotec), as per manufacturer’s instructions and their viability confirmed by Trypan blue staining.

### *In vitro* neutrophil viability through LDH release

In a tissue culture-treated 96-well plate, 2 × 10^5^ isolated mouse bone marrow neutrophils in 200 μl of DMEM + 10% (v/v) heat-inactivated FBS + 1% (v/v) penicillin/streptomycin were incubated for 6 or 24 hours. Culture supernatants were collected and cells were lysed using 1x lysis buffer (Promega). 10x lysis buffer was added prior to supernatant collection as a positive control. Lactose dehydrogenase activity in both the culture supernatants and cell lysates were then determined using the CytoTox Non-Radioactive Cytotoxicity Assay (Promega), as per manufacturer’s instructions.

### Neutrophil ROS activity

Following isolation of neutrophils from bone marrow, cells were incubated with 5 μM chloromethyl-dichlorodihydrofluorescein diacetate (CM-H_2_DCFDA) (Thermo) in PBS for 45 mins at 37°C, 5% CO_2_. Cells were then washed and resuspended in RPMI without phenol red, supplemented with 10% (v/v) FBS and 1% (v/v) penicillin/streptomycin. Cells were then plated at a concentration of 2 × 10^5^ cells / well and incubated in the presence or absence of 2 μM PMA. Fluorescence was measured at the indicated time points using an excitation of 492 nm and an emission of 527 nm.

### Neutrophil-mediated fungal growth inhibition

*A*. *fumigatus* Af293 was grown for 6 hours in DMEM + 10% (v/v) heat-inactivated FBS + 1% (v/v) penicillin/streptomycin at a concentration of 3 × 10^3^ conidia / well. Isolated mouse bone marrow neutrophils were co-incubated with hyphae for 16 hours at a concentration of 10^5^ or 2 × 10^5^ cells / well (MOI 1:33 or 1:66, respectively). The resulting hyphal growth was then measured by staining with a 1 mg/ml solution of calcofluor white, and measuring the fluorescence with an excitation of 340 nm and an emission at 440 nm. Data is reported relative to the growth of fungi not incubated with neutrophils (untreated, UT).

### Adoptive transfer of neutrophils

Mouse bone marrow neutrophils were isolated as above in PBS and their purity and viability confirmed by flow cytometry analysis. Twenty-four hours post infection, neutrophils were administered to mice by lateral tail vein injection (10^6^ live neutrophils in 200 μl). At 36 hours post infection (12 hours post neutrophil administration), mice were euthanized and their blood, lungs and BAL fluid were collected and processed as above. Samples were stained as above with the following antibodies: CD45.1-FITC, CD45.2-APC-Cy7, CD11b-PE-CF594, CD11c-APC, Ly6G-PE, Ly6C-Alexa Fluor 700, and SiglecF-brilliant violet 421. Samples were analyzed by flow cytometry. Neutrophils were defined as Ly6G^+^ Ly6C^mid^ CD11c^−^ CD11b^+^ before being gated as either CD45.1^+^ CD45.2^−^ or CD45.1^−^ CD45.2^+^. For gating strategy and absolute numbers of donor neutrophils detected in the blood, lung tissue and BAL fluid, see S9 Fig.

### Pulmonary intravital imaging

Pulmonary intravital microscopy was performed as previously described [49]. Mice were infected with Af293 expressing mRFP1 as above and at 16 hours post infection were anaesthetized with xylazine hydrochloride and ketamine hydrochloride (10 mg and 200 mg per kg body weight, respectively) administered intraperitoneally. The mice were cannulated via the right jugular vein using a catheter and tracheotomised to be put on mechanical ventilation. The lung was exposed via lateral thoracotomy, and stabilized for imaging using a vacuum-chamber fitted with a glass slide. To visualize neutrophils, anti-Ly6G (clone 1A8, BioLegend) conjugated to Brilliant Violet 421 was administered intravenously (1.5 μg per mouse) 5 to 10 mins prior to imaging. Imaging was performed on a resonant-scanner confocal microscope (Leica SP8), and the resulting time-lapse videos were analyzed with either Leica LAF or Volocity software. Firm adhesion is characterized as a neutrophil that remained stationary and non-motile for at least 30 seconds, with % adhesion representing the proportion of neutrophils observed that were classified as having this characteristic [50].

### Neutrophil live cell imaging assay

Neutrophils were purified from the blood of healthy volunteers, as previously described [40]. Neutrophil motility assays were performed as previously described [51,52]. Briefly, purified human neutrophils were added to collagen type I-coated (50μg/ml) wells of Nunc Lab-Tek II chambered coverglass and allowed to adhere for 1 hour in RPMI1640 media at room temperature for 1 hour, then transferred to an environmental chamber (Live Cell Instrument, Korea) on the stage of the microscope (Quorum Technologies) as previously described [52]. Cells were maintained at 37°C, 7.5% CO_2_ with 80% humidity. PBS, recombinant galectin-3 or galectin-3 CRD were added to a final concentration of 1 μM, and cells were imaged using the Quorum spinning disc confocal system, composed of an Olympus microscope under the control of MetaMorph software (Molecular Devices), together with in-house software that can concurrently create movies [53]. Twenty-four fields of view per condition were acquired using a 10× objective (Olympus Uplsapo Super Apochromat, numerical aperture 0.4) and a near-infrared light-emitting diode (pE-100, 740 nm; CoolLED, Andover, United Kingdom) collecting differential interference contrast images, every 5 mins overnight. Images were magnified using a 1.5× coupler (Quorum Technologies) inserted into the light path to the CCD cameras, to create images equivalent to 15× magnification. Cells were individually tracked and their distances travelled at each time point were calculated via in-house software through the positions of the cells at each time point [53]. The average speed of each cell during the experiment was then calculated, and normalized to the PBS group.

### Statistical analyses

All graphs were generated and statistical analyses were performed using Prism v6.0 or v8.1 (GraphPad Software). All data represent distinct measurements, unless otherwise noted. Statistical significance calculated as indicated. All data presented as the mean ± standard error of mean (SEM), unless otherwise noted.

## Supporting information

S1 Fig Intracellular galectin-3 is broadly expressed in the lungs of naïve and *A*. *fumigatus-*infected mice. **(A)** Gating strategy for differential cell staining flow cytometry of mouse lung digests. The definition of positive/negative gates determined from “fluorescence minus one” (FMO) controls. **(B)** Representative histograms of intracellular galectin-3 for each cell type in infected galectin-3 deficient (Gal3^-/-^ infected), uninfected C57BL/6 (B6 uninfected), and infected C57BL/6 (B6 infected) mice. **(C)** The galectin-3 geometric mean fluorescent intensity (MFI), **(D)** galectin-3^+^ proportions, and **(E)** quantification of the cell types indicated in the lung digests of the C57BL/6 mice. *n* = 8 Gal3^-/-^ infected, 10 B6 uninfected and 12 B6 infected from 2 independent experiments. 2-way ANOVA with Sidak’s multiple comparison post-test.
(PDF)

S2 Fig Mice do not exhibit sex-dependent survival or fungal burden difference during pulmonary *A*. *fumigatus* infection. Stratification by sex of **(A)** survival and (**B**) fungal burden of the indicated stains of mice following *A*. *fumigatus* Af293 infection. For survival experiments, *n* = 18 male and 18 female C57BL/6, and 18 male and 17 female galectin-3 deficient mice from 4 independent experiments. For fungal burden experiments, *n* = 11 male and 10 female infected C57BL/6, and 11 male and 9 female infected galectin-3 deficient mice from 3 independent experiments. Mantel-Cox log rank test for survival experiments, and 1-way ANOVA with Sidak’s multiple comparison post-test for fungal burden.
(PDF)

S3 Fig Galectin-3 binds to the surface of *A*. *fumigatus* conidia. **(A)** Gating strategy for measuring galectin-3 staining on the surface of resting and swollen conidia. **(B)** Af293 resting and swollen conidia stained with recombinant galectin-3 and analyzed by flow cytometry. Representative histograms from 4 independent experiments. **(C)** Geometric mean fluorescent intensity of galectin-3 staining of 4 independent experiments. *: *p* < 0.05 compared to both unstained (U/S) and fluorescent streptavidin (2nd) controls; 1-way ANOVA with Tukey’s multiple comparison test.
(PDF)

S4 Fig Additional cellular analyses of the lung tissue and airways during pulmonary *A*. *fumigatus* infection. **(A)** Quantification of eosinophils, inflammatory monocytes, and alveolar macrophages in the lung digest and **(B)** BAL fluid of immunocompetent C57BL/6 and galectin-3 deficient mice 36 hours post-infection. For quantification of eosinophils and alveolar macrophages in lung digests, *n* = 12 uninfected and 19 infected C57BL/6, and 11 uninfected and 18 infected galectin-3 deficient mice from 3 independent experiments. For quantification of eosinophils and alveolar macrophages in BAL fluid, *n* = 19 uninfected and 33 infected C57BL/6, and 18 uninfected and 32 infected galectin-3 deficient mice from 5 independent experiments. For quantification of inflammatory monocytes, *n* = 9 uninfected and 12 infected C57BL/6, and 8 uninfected and 12 infected galectin-3 deficient mice from 2 independent experiments for both lung digest and BAL fluid. 2-way ANOVA with Sidak’s multiple comparison test.
(PDF)

S5 Fig Cytokines and chemokines downregulated in the bronchoalveolar lavage fluid of galectin-3 deficient mice compared to C57BL/6 mice during pulmonary *A*. *fumigatus* infection. Cytokine concentrations were measured in the BAL fluid of mice 36 hours post pulmonary *A*. *fumigatus* infection. *n* = 8 uninfected and 14 infected C57BL/6, and 7 uninfected and 13 infected galectin-3 deficient mice from 2 independent experiments. ND: non-detect. 2-way ANOVA with Sidak’s multiple comparison test.
(PDF)

S6 Fig Galectin-3 deficient mice exhibit normal pulmonary vasculature. **(A)** Flow cytometry gating strategy for endothelial cells. Representative plots from infected C57BL/6 lung digest. The definition of positive/negative gates determined from FMO controls. **(B)** Staining intensity of C57BL/6 and galectin-3 deficient endothelial cells from for the indicated surface markers were analyzed by flow cytometry. *n* = 15 infected C57BL/6 mice and 18 infected galectin-3 deficient mice from three independent experiments. 2-way ANOVA with Sidak’s multiple comparison test.
(PDF)

S7 Fig Flow cytometry gating strategy for the pulmonary innate immune cell compartment. Representative plots from infected C57BL/6 lung digest. The definition of positive/negative gates determined from FMO controls.
(PDF)

S8 Fig Flow cytometry gating strategy for the vascular staining of the pulmonary innate immune cell compartment. Representative plots from infected C57BL/6 lung digest. The definition of positive/negative gates determined from FMO controls.
(PDF)

S9 Fig Flow cytometry gating strategy and absolute quantification for the adoptive transfer of bone marrow-isolated neutrophils. **(A)** Representative plots from infected C57BL/6 lung digest. The definition of positive/negative gates determined from FMO controls. **(B)** Absolute numbers of donor neutrophils detected in the blood, lung tissue and BAL fluid from Fig 5E and 5G.
(PDF)

S1 Table Fluorescent antibodies used in this study. (PDF)

S1 Movie Neutrophils colocalize and occupy the same focal plane as *A*. *fumigatus* in the lungs of infected wild-type mice. (MP4)
