# Supplementary figures and images for: Galectin-3 enhances neutrophil motility and extravasation into the airways during Aspergillus fumigatus infection
Source: PLoS Pathog. 2020 Aug 4;16(8):e1008741. doi: 10.1371/journal.ppat.1008741 (PMC7428289; doi:10.1371/journal.ppat.1008741)

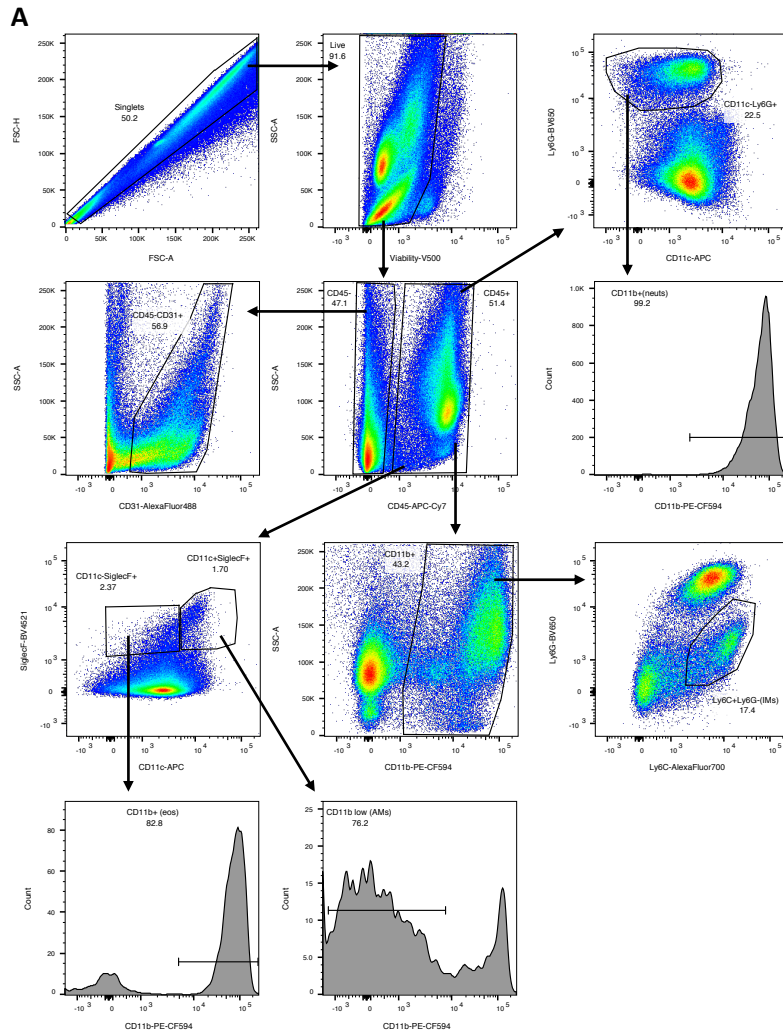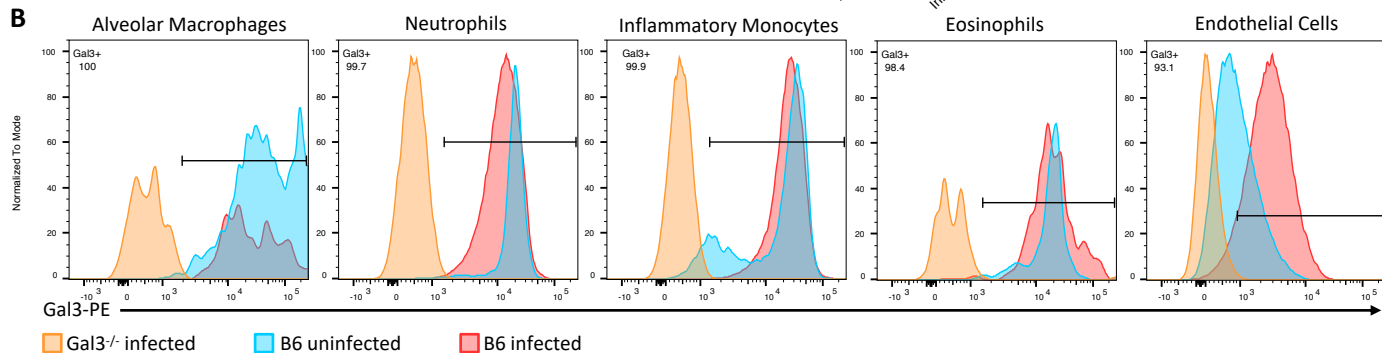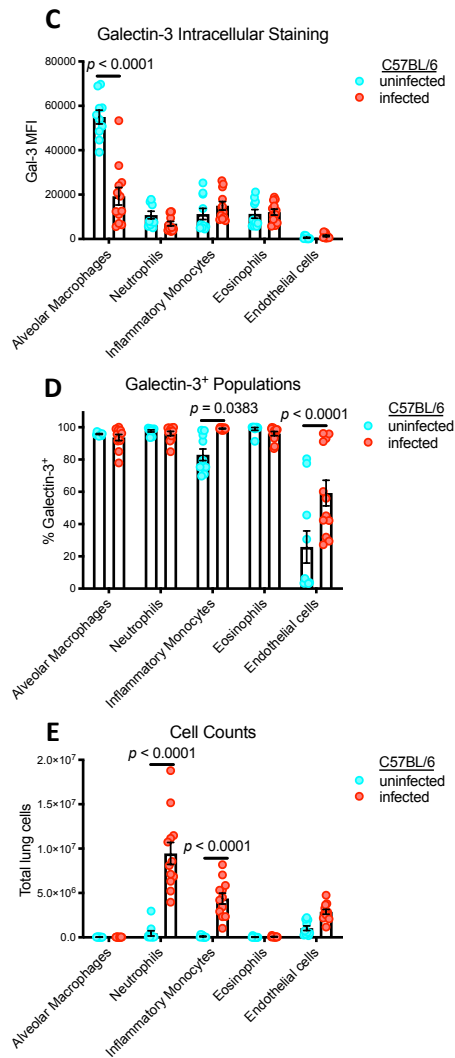

Supplement: S1 Fig — (A) Gating strategy for differential cell staining flow cytometry of mouse lung digests. The definition of positive/negative gates determined from “fluorescence minus one” (FMO) controls. (B) Representative histograms of intracellular galectin-3 for each cell type in infected galectin-3 deficient (Gal3-/- infected), uninfected C57BL/6 (B6 uninfected), and infected C57BL/6 (B6 infected) mice. (C) The galectin-3 geometric mean fluorescent intensity (MFI), (D) galectin-3+ proportions, and (E) quantification of the cell types indicated in the lung digests of the C57BL/6 mice. n = 8 Gal3-/- infected, 10 B6 uninfected and 12 B6 infected from 2 independent experiments. 2-way ANOVA with Sidak’s multiple comparison post-test. (PDF) [file ppat.1008741.s001.pdf]

**A**

Af293

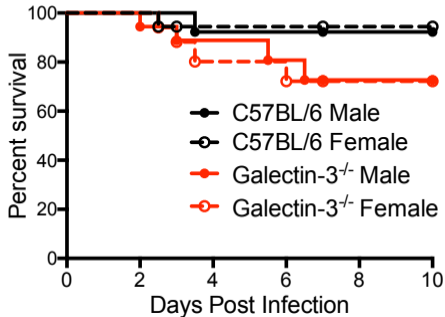**B**

Af293 Fungal Burden

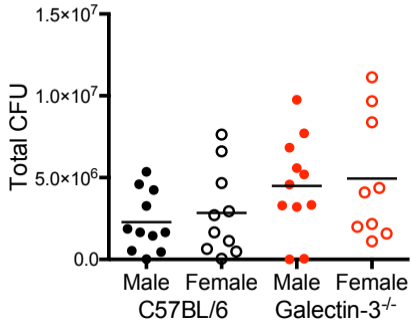

Supplement: S2 Fig — Stratification by sex of (A) survival and (B) fungal burden of the indicated stains of mice following A. fumigatus Af293 infection. For survival experiments, n = 18 male and 18 female C57BL/6, and 18 male and 17 female galectin-3 deficient mice from 4 independent experiments. For fungal burden experiments, n = 11 male and 10 female infected C57BL/6, and 11 male and 9 female infected galectin-3 deficient mice from 3 independent experiments. Mantel-Cox log rank test for survival experiments, and 1-way ANOVA with Sidak’s multiple comparison post-test for fungal burden. (PDF) [file ppat.1008741.s002.pdf]

**A**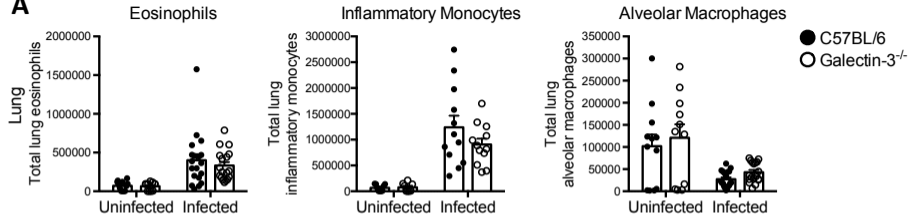**B**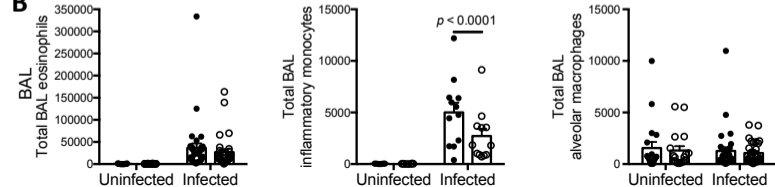

Supplement: S4 Fig — (A) Quantification of eosinophils, inflammatory monocytes, and alveolar macrophages in the lung digest and (B) BAL fluid of immunocompetent C57BL/6 and galectin-3 deficient mice 36 hours post-infection. For quantification of eosinophils and alveolar macrophages in lung digests, n = 12 uninfected and 19 infected C57BL/6, and 11 uninfected and 18 infected galectin-3 deficient mice from 3 independent experiments. For quantification of eosinophils and alveolar macrophages in BAL fluid, n = 19 uninfected and 33 infected C57BL/6, and 18 uninfected and 32 infected galectin-3 deficient mice from 5 independent experiments. For quantification of inflammatory monocytes, n = 9 uninfected and 12 infected C57BL/6, and 8 uninfected and 12 infected galectin-3 deficient mice from 2 independent experiments for both lung digest and BAL fluid. 2-way ANOVA with Sidak’s multiple comparison test. (PDF) [file ppat.1008741.s004.pdf]

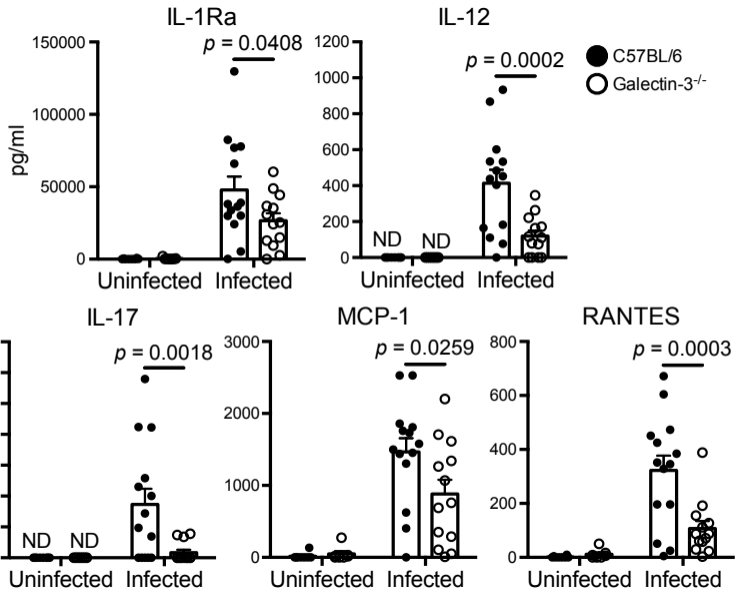

Supplement: S5 Fig — Cytokine concentrations were measured in the BAL fluid of mice 36 hours post pulmonary A. fumigatus infection. n = 8 uninfected and 14 infected C57BL/6, and 7 uninfected and 13 infected galectin-3 deficient mice from 2 independent experiments. ND: non-detect. 2-way ANOVA with Sidak’s multiple comparison test. (PDF) [file ppat.1008741.s005.pdf]

**A**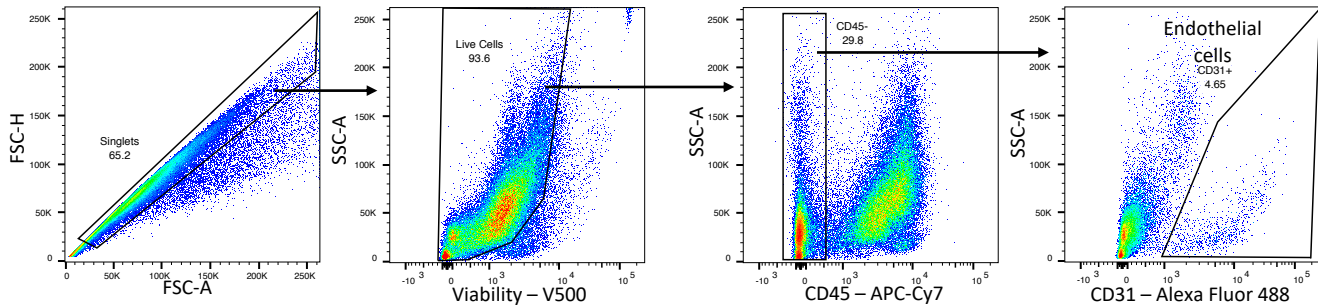**B**

### Endothelial Cell Surface Protein Expression

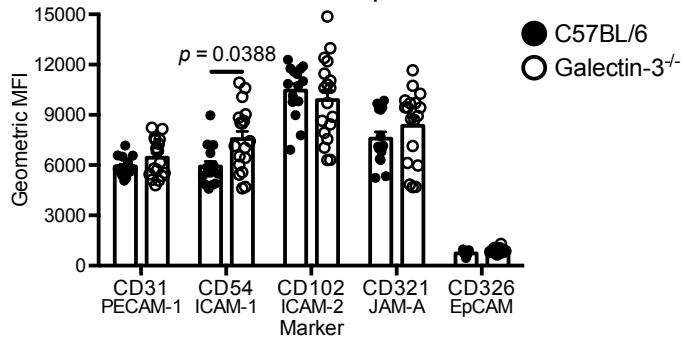

Supplement: S6 Fig — (A) Flow cytometry gating strategy for endothelial cells. Representative plots from infected C57BL/6 lung digest. The definition of positive/negative gates determined from FMO controls. (B) Staining intensity of C57BL/6 and galectin-3 deficient endothelial cells from for the indicated surface markers were analyzed by flow cytometry. n = 15 infected C57BL/6 mice and 18 infected galectin-3 deficient mice from three independent experiments. 2-way ANOVA with Sidak’s multiple comparison test. (PDF) [file ppat.1008741.s006.pdf]

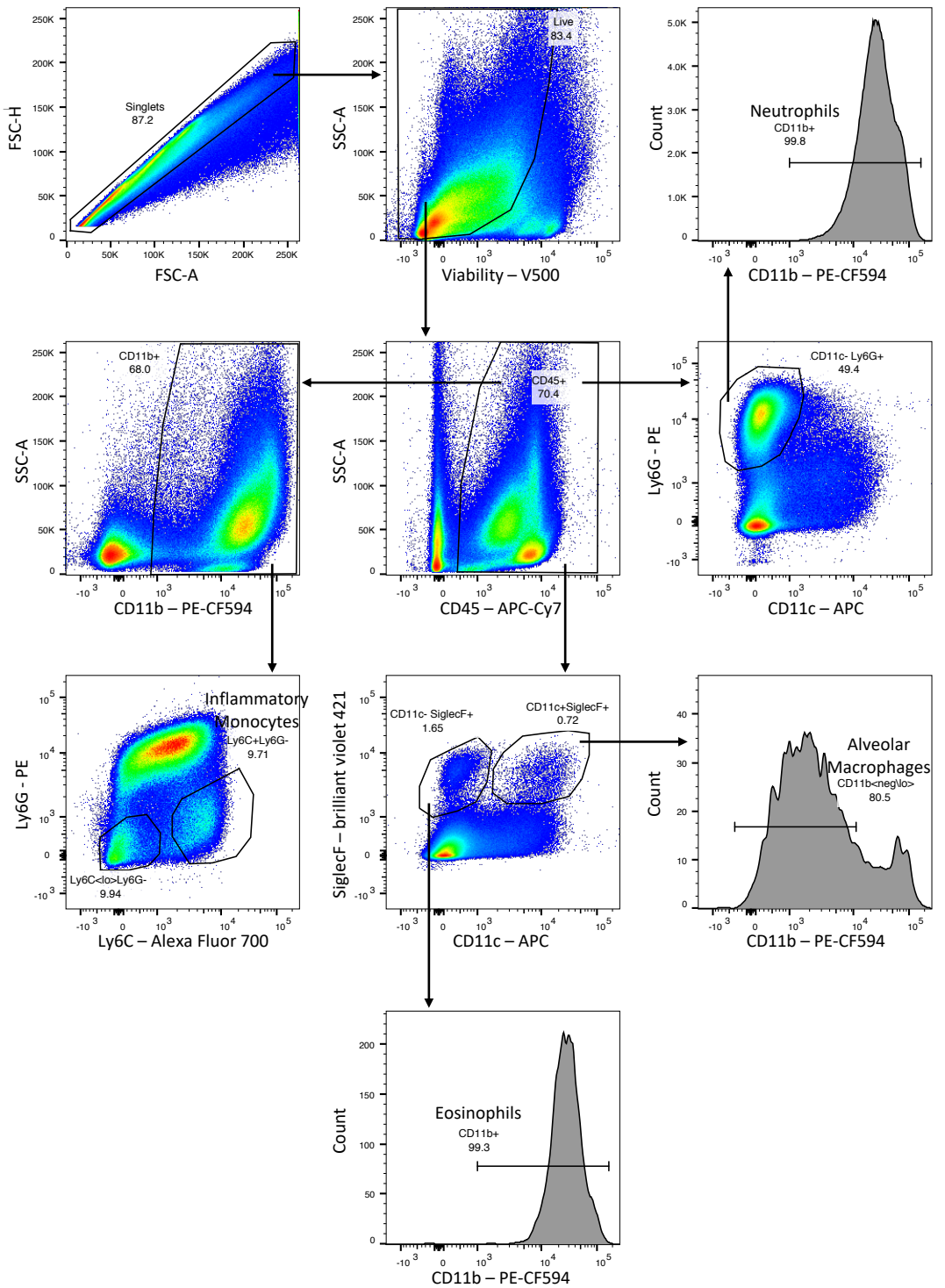

Supplement: S7 Fig — Representative plots from infected C57BL/6 lung digest. The definition of positive/negative gates determined from FMO controls. (PDF) [file ppat.1008741.s007.pdf]

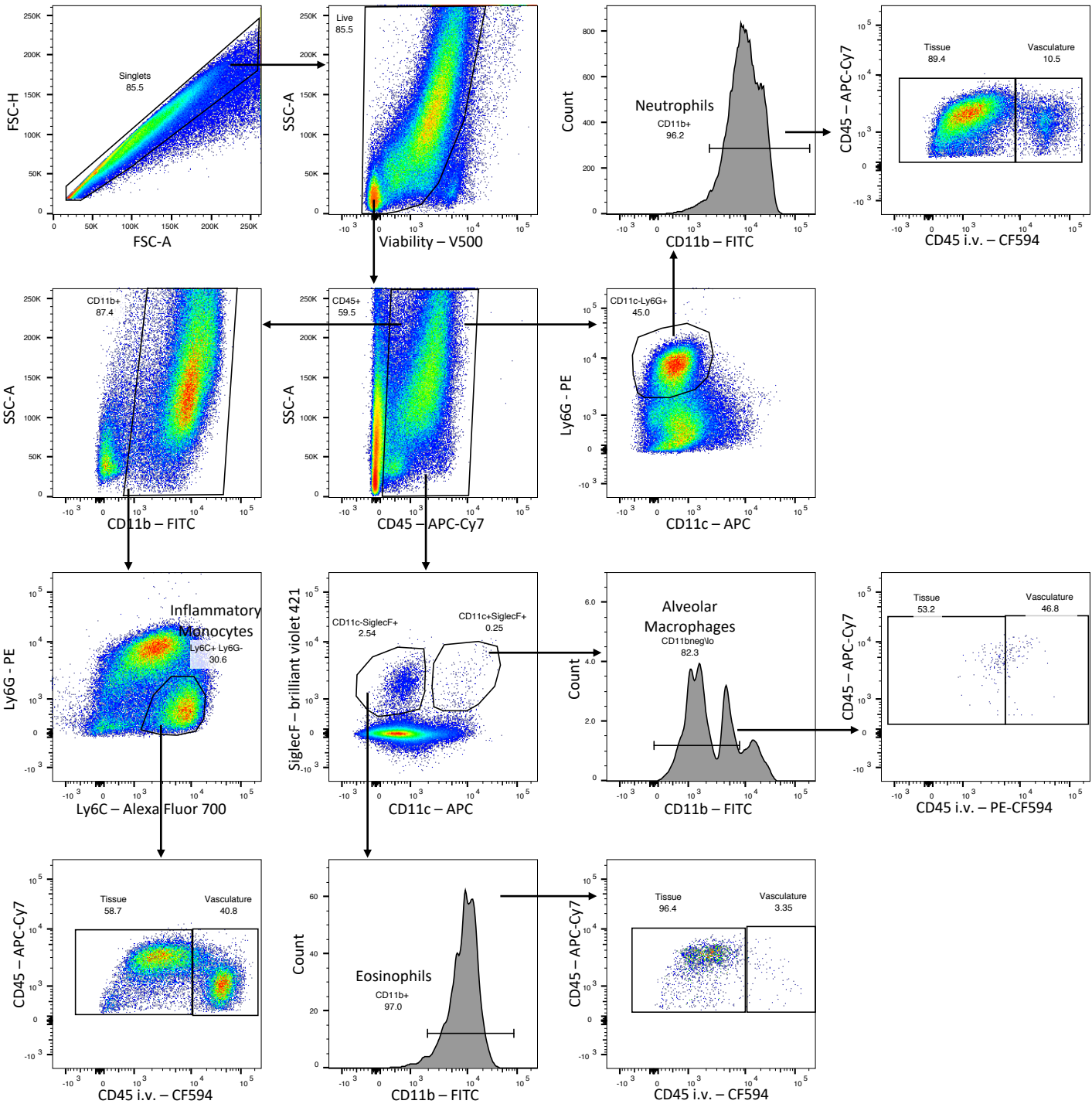

Supplement: S8 Fig — Representative plots from infected C57BL/6 lung digest. The definition of positive/negative gates determined from FMO controls. (PDF) [file ppat.1008741.s008.pdf]

**A**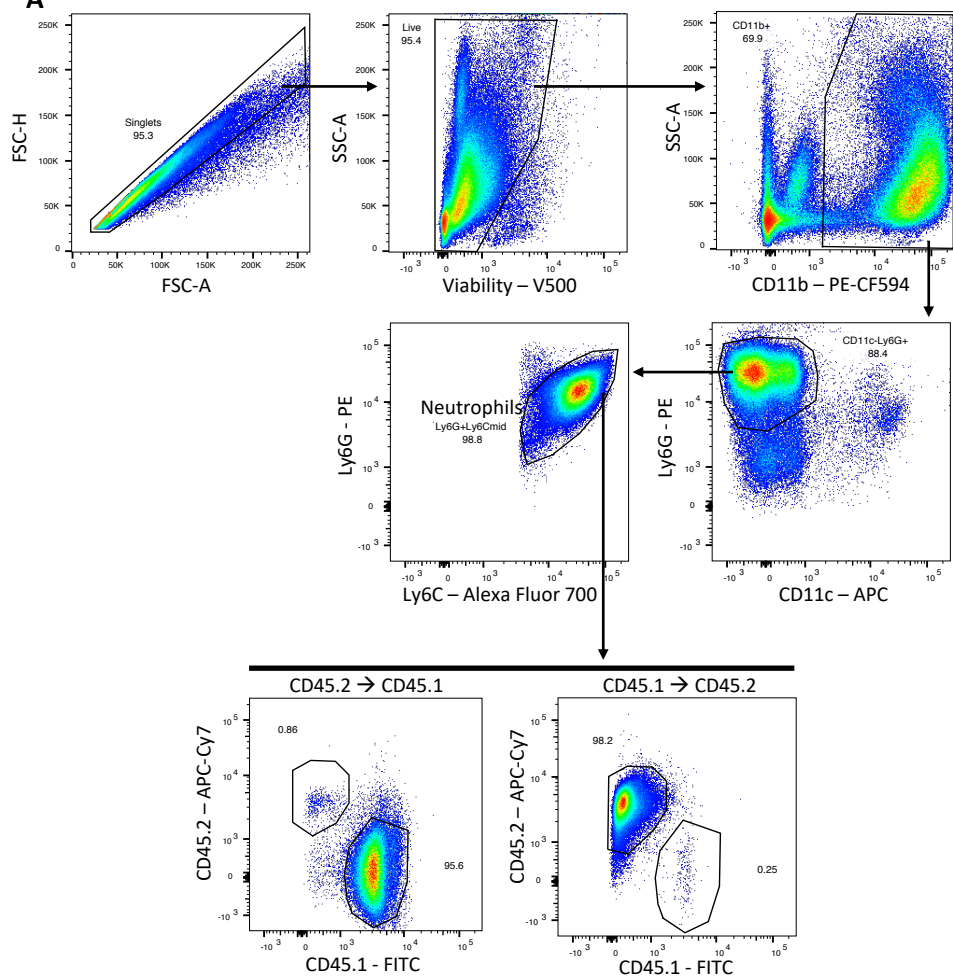**B**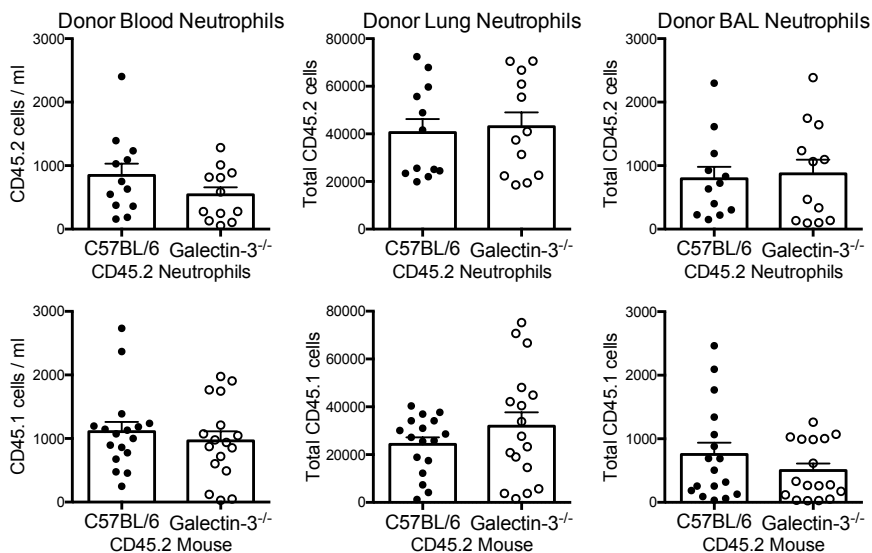

Supplement: S9 Fig — (A) Representative plots from infected C57BL/6 lung digest. The definition of positive/negative gates determined from FMO controls. (B) Absolute numbers of donor neutrophils detected in the blood, lung tissue and BAL fluid from Fig 5E and 5G. (PDF) [file ppat.1008741.s009.pdf]
