# Supplementary material for: Galectin-3 enhances neutrophil motility and extravasation into the airways during Aspergillus fumigatus infection
Source: PLoS Pathog. 2020 Aug 4;16(8):e1008741. doi: 10.1371/journal.ppat.1008741 (PMC7428289; doi:10.1371/journal.ppat.1008741)
Supplement: S3 Fig — (A) Gating strategy for measuring galectin-3 staining on the surface of resting and swollen conidia. (B) Af293 resting and swollen conidia stained with recombinant galectin-3 and analyzed by flow cytometry. Representative histograms from 4 independent experiments. (C) Geometric mean fluorescent intensity of galectin-3 staining of 4 independent experiments. *: p < 0.05 compared to both unstained (U/S) and fluorescent streptavidin (2nd) controls; 1-way ANOVA with Tukey’s multiple comparison test. (PDF) [file ppat.1008741.s003.pdf]

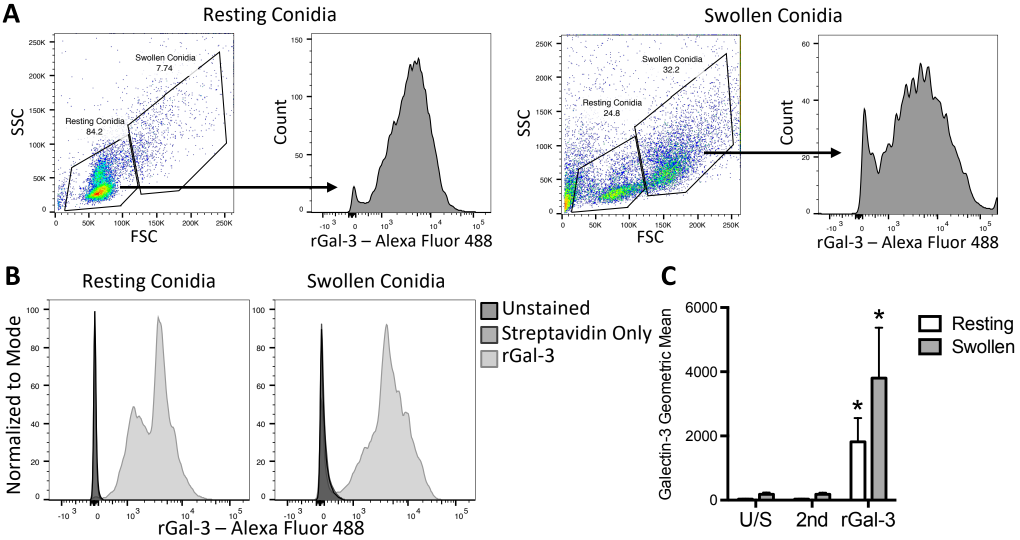
A
Resting Conidia
Swollen Conidia
7.74
Resting Conidia
84.2
SSC
FSC
Count
rGal-3 – Alexa Fluor 488
Swollen Conidia
Swollen Conidia
32.2
Resting Conidia
24.8
B
Resting Conidia
Swollen Conidia
Normalized to Mode
Unstained
Streptavidin Only
rGal-3
rGal-3 – Alexa Fluor 488
C
Galectin-3 Geometric Mean
Resting
Swollen
U/S
2nd
rGal-3
*
*
