# Supplementary material for: Galectin-3 enhances neutrophil motility and extravasation into the airways during Aspergillus fumigatus infection
Source: PLoS Pathog. 2020 Aug 4;16(8):e1008741. doi: 10.1371/journal.ppat.1008741 (PMC7428289; doi:10.1371/journal.ppat.1008741)
Supplement: S1 Table — (PDF) [file ppat.1008741.s010.pdf]

Marker	Fluorophore	Clone	Supplier	CAT#	Lot#
CD11b	phycoerythrin (PE)-CF594	M1/70	BD	562287	7054816
CD11c	allophycocyanin (APC)	HL3	BD	550261	7179633
CD11b	fluorescein isothiocyanate (FITC)	M1/70	Biolegend	101206	B160103
CD31	Alexa Fluor®488	390	BD	563607	7278830
CD45	APC-Cy7	30-F11	BD	557659	7215837
CD45	PE-CF594	30-F11	BD	562420	8281746
CD45.1	FITC	A20	BD	553775	7191533
CD45.2	APC-Cy7	104	BD	560694	7306655
CD54	brilliant ultraviolet 395	3E2	BD	740222	9024929
CD102	brilliant violet 605	3C4(mlC2/4)	BD	740346	9024976
CD321	brilliant violet 711	H202-106	BD	745405	9024933
CD326	brilliant violet 786	G8.8	BD	740958	7101853
SiglecF	brilliant violet 421	E50-2440	BD	562681	8004532
Ly6G	PE	1A8	BD	551461	5362993
Ly6C	Alexa Fluor®700	AL-21	BD	561237	8186578
Ly6G	brilliant violet 421	1A8	Biolegend	127628	
Ly6G	brilliant violet 650	1A8	BD	740554	6152679
Galectin-3	PE	M3/38	Invitrogen	12-5301-82	2009791
